# Evaluation of Reference Genes for Accurate Normalization of Gene Expression for Real Time-Quantitative PCR in *Pyrus pyrifolia* Using Different Tissue Samples and Seasonal Conditions

**DOI:** 10.1371/journal.pone.0086492

**Published:** 2014-01-22

**Authors:** Tsuyoshi Imai, Benjamin E. Ubi, Takanori Saito, Takaya Moriguchi

**Affiliations:** 1 Plant Physiology and Fruit Chemistry Division, NARO Institute of Fruit Tree Science, Tsukuba, Ibaraki, Japan; 2 Biotechnology Research & Development Centre, Ebonyi State University, Abakaliki, Ebonyi State, Nigeria; 3 Graduate School of Life and Environmental Sciences, University of Tsukuba, Tsukuba, Ibaraki, Japan; Naval Research Laboratory, United States of America

## Abstract

We have evaluated suitable reference genes for real time (RT)-quantitative PCR (qPCR) analysis in Japanese pear (*Pyrus pyrifolia*). We tested most frequently used genes in the literature such as *β*-*Tubulin*, *Histone H3*, *Actin*, *Elongation factor-1α*, *Glyceraldehyde-3-phosphate dehydrogenase*, together with newly added genes *Annexin*, *SAND* and *TIP41*. A total of 17 primer combinations for these eight genes were evaluated using cDNAs synthesized from 16 tissue samples from four groups, namely: flower bud, flower organ, fruit flesh and fruit skin. Gene expression stabilities were analyzed using geNorm and NormFinder software packages or by ΔCt method. geNorm analysis indicated three best performing genes as being sufficient for reliable normalization of RT-qPCR data. Suitable reference genes were different among sample groups, suggesting the importance of validation of gene expression stability of reference genes in the samples of interest. Ranking of stability was basically similar between geNorm and NormFinder, suggesting usefulness of these programs based on different algorithms. ΔCt method suggested somewhat different results in some groups such as flower organ or fruit skin; though the overall results were in good correlation with geNorm or NormFinder. Gene expression of two cold-inducible genes *PpCBF2* and *PpCBF4* were quantified using the three most and the three least stable reference genes suggested by geNorm. Although normalized quantities were different between them, the relative quantities within a group of samples were similar even when the least stable reference genes were used. Our data suggested that using the geometric mean value of three reference genes for normalization is quite a reliable approach to evaluating gene expression by RT-qPCR. We propose that the initial evaluation of gene expression stability by ΔCt method, and subsequent evaluation by geNorm or NormFinder for limited number of superior gene candidates will be a practical way of finding out reliable reference genes.

## Introduction

Gene expression analysis is an increasingly important strategy towards advancing our understanding of the complex signaling and metabolic pathways underlying developmental and cellular processes in biological organisms including plants. Northern blotting, ribonuclease protection assay, reverse transcription-polymerase chain reaction (RT-PCR), semi-quantitative RT-PCR, DNA microarrays and real time-quantitative PCR (RT-qPCR) have all been applied in the analysis of gene expression [1]. Among these methods, however, the recently developed techniques of microarray and RT-qPCR have relatively gained much prominence and wider applicability for the quantification of gene expression, owing to their inherent advantages of speed, high-throughput and automation potential. While DNA microarray technology allows the parallel monitoring of a large set of genes in a single experiment involving two differentially labeled RNA populations, RT-qPCR enables simultaneous quantification of gene expression in a large sample set, but for a limited number of genes [2], [3].

The RT-qPCR technique, which has a long history of application in the medical sciences [4], has now been widely applied for the analysis of gene expression in plants [3]. It provides a powerful tool for quantifying changes in gene expression, as well as its usefulness as the standard method for the validation of high-throughput or microarray data, due to its ability to integrate efficiency in the techniques for signal detection with improved sensitivity and specificity [5], [6], [7]. Recently, the limitation of RT-qPCR in its ability to assess only a limited number of genes have been overcome with the development of the microfluidic technology that allows for high-throughput measurement of gene expression using dynamic arrays [8]. This microfluidic technology, which allows 9,216 simultaneous real-time PCR transcript quantification per single run has now been extended to studies in plants such as *Eucalyptus* [9]. Another way to accomplish high throughput RT-qPCR has been proposed by using nanoliter plate with 3,072 sample holes [10]. Moreover, new generation sequencing data is currently expanding in many plant species and RT-qPCR provides a reliable method for validating such huge amount of RNA-seq data.

Right from the older method of Northern blotting, reliable transcript normalization with internal standards ‘housekeeping genes’ has been of crucial consideration to ensure robustness and accuracy of the technique [1], [11]. Prior to the genomic era, housekeeping genes (i.e. genes believed to be required for basic cellular metabolism and maintenance) such as *18S rRNA*, *Glyceraldehyde-3-phosphate dehydrogenase* (*GAP*), *β-* and *γ-Actin*, *Elongation factor-1α* (*EF1a*), *α-* and *β-Tubulin* (*bTUB*), and *Ubiquitin* (*UBI*) were commonly used for transcript normalization. These ‘housekeeping genes’ were used in plant studies because they were thought to be uniformly expressed across different experimental conditions (treatments, tissues and developmental stages). However, the growing application of the highly sensitive RT-qPCR method has indicated that the expression of these so-called housekeeping genes varies under a given set of experimental conditions and their systematic use without prior validation can result in the misinterpretation of the data [12]. Thus, among the golden rules of the RT-qPCR technique [13], transcript level normalization with the ideal reference gene(s) is a critical factor. The term ‘reference genes’ therefore should apply to genes whose stable expressions have been experimentally validated in the respective species and tissues, under the given set of experimental conditions. Where no single gene is found to show such stable expression, it is recommended that two or more such validated genes be used to ensure more accurate transcript normalization [2], [12], [14], [15]. Several algorithms including geNorm [2], NormFinder [16], BestKeeper [17], ΔCt [18], qBasePlus [19], RefFinder [20], as well as single-factor analysis of variance (ANOVA) and linear regression analysis [14] have been used to evaluate the expression stability of reference genes. In recent times, studies on reference genes stability have been reported in plants including the model, crop and woody species [6], [9], [15], [21], [22], [23], [24], though these studies are relatively few compared to similar studies in animals or humans. The results of these studies suggested that expression stability of reference gene candidates varies depending on the plant source materials tested and a need for multiple reference genes for accurate evaluation [2], [15]. A detailed verification of microarray expression data by RT-qPCR in *Arabidopsis* [6] discovered some new genes with quite stable expression such as *SAND family protein* (*SAND*; *At2g28390*), *TIP41-like* (*TIP*; *At4g34270*) and *unknown expressed protein* (*At4g26410*).

Japanese pear (*Pyrus pyrifolia* Nakai) is a member of the Rosaceae family belonging to the subfamily Pyroideae, which constitute an economically-significant group of fleshy fruit species important for human diet, dietary diversity and ornamental beauty. The Pyroideae subfamily (to which pear and apple belong) are characterized by the pome fruits which is made up of complex fruiting organs with different expanded tissues derived from the floral tube [25]. Research towards understanding the molecular mechanisms underlying the diverse regulatory pathways in this important fruit tree species involving the use of RT-qPCR have increasingly expanded in the last decade. Such published studies include the identification of dormancy associated *MADS-box* genes [26], transcriptome analysis during dormancy transitional phases [27], [28], skin pigmentation with R2R3-type *MYB* gene [29], etc., in which *Actin* (*ACT*), *EF1a*, *and Histone H3* (*HIS*) were used as reference genes, to quantify the relative transcript expression levels without any experimental confirmation of stability in their gene expressions. To the best of our knowledge, the systematic validation of reference genes suitable for RT-qPCR gene expression analysis in *P. pyrifolia* has yet been reported.

In the present study, we validated eight candidate internal reference genes representing at least 15 gene loci by evaluating their stability of gene expression using a diverse set of *P. pyrifolia* tissue samples representing different environmental conditions, tissue types and developmental stages. Amplification of sequences across stop codons containing 3′-end of coding sequences (CDS) and neighboring 3′-untranslated region (UTR) revealed differential expression pattern among homologous genes. We have shown in this study that gene expression stabilities are different among the sample types tested; thus, experimental validation for normalizing RT-qPCR data is needful. We propose that three best performing genes judged by geNorm are sufficient for reliable evaluation of RT-qPCR data. Furthermore, expression analysis of two cold-inducible target genes, *PpCBF2* and *PpCBF4* {C-repeat/(dehydration−/low temperature-responsive element) Binding Factor}, were presented to demonstrate the utility of these set of validated reference genes.

## Results

### Selection and 3′-Rapid Amplification of cDNA Ends (RACE) Cloning of Reference and Target Genes

We selected five most frequently used genes for RT-qPCR analysis: *bTUB*, *HIS*, *EF1a*, *GAP* and *ACT*. In addition, we employed three new genes *Annexin* (*ANX*), *SAND* and *TIP*. Annexin is a group of calcium-dependent phospholipid-binding proteins involved in membrane organization and trafficking, exo- and/or endo-cytosis [30], [31]. *SAND* and *TIP* homologues were found to be most stably expressed genes in *Arabidopsis* [6]. SAND is a DNA-binding domain found in many nuclear proteins and classified as PF01342 in protein family database Pfam (http://pfam.janelia.org/). SAND protein homologues are expressed in almost all eukaryotic cells. Functional studies in yeast coupled with protein characterization *in silico* [32] suggested its roles in vacuoles or lysosomes. Although genes for SAND homologous proteins are isolated and their gene expression stabilities evaluated using plant cells or tissues [6], [9], [21], [22], [33], [34], no functional studies have been done. TIP41 (TAP42-interacting protein of 41 kDa) is an accessory protein regulating the activity of protein phosphatase through interaction with a phosphatase inhibitor protein TAP42 in yeast [35]. A mammalian TIP41 homologue, TIPRL regulates the activity of protein phosphatase 2A through direct interaction in human cells [36]. TIP41 acts as a negative regulator of target of rapamycin complex in yeast [35], whereas a positive effect was observed in human cells [36]. *TIP41* gene homologues seem to be expressed universally in plant tissues [6], [21], [22], [34], [37]; however, no functional analysis has been reported to date. The functions of the candidate reference genes are shown in Table S1.

Based on the publicly available apple EST and genomic sequences, 3′-RACE primers (Table 1) were designed on a relatively conserved region of the genes. In most cases, amplified fragments with the expected sizes were obtained with touchdown PCR. In case of ambiguous bands in the first PCR product, nested 3′-RACE primer was used to re-amplify the product from one-fiftieth diluted first round RACE product. Based on the sequence data of the cloned amplified product, cDNA from more than two gene loci were obtained for *bTUB*, *HIS*, *EF1a*, *ACT* and *GAP* (Table 1). Amino acid identities among homologous genes were more than 89% (Table S2), suggesting functional identity of the gene product. Nucleotide sequences were highly conserved in the protein coding region, while significant differences were revealed in the 3′-UTR (Table S2). Quite high nucleotide identity between *Annexin#1* and *#2* was observed in the protein coding sequence, while it was low in the first 150 nt of 3′-UTR (Table S2). This probably indicates that the two clones are splicing variants rather than representing different gene loci. Based on the relatively low value (from 79 to 96%) of nucleotide identity in CDS, other clones are probably derived from different gene loci. We obtained two *CBF* homologues *PpCBF2* and *PpCBF4*. Amino acid identity between *Pyrus* and *Malus* sequences are more than 95% between corresponding gene products (e.g. MdCBF2 vs. PpCBF2), while that between gene homologues within a species (e.g. PpCBF2 vs. PpCBF4) was 74–80% (Table S3). For 3′-UTR sequences, nucleotide identity were relatively lower, around 60 to 70% range, with exceptionally high value (90%) between *PpCBF4*_*utr* vs. *MdCBF4*_*utr* (Table S3).

**Table 1:** 3′-RACE cloning of candidate reference genes and cold-inducible genes.

Symbol	Gene name	CDS (nt)	3′-UTR^a^(nt)	Number ofhomologousgenes	3′-RACE primer(Amino acid residue)	3′-RACEclone name	GenBank#	*Arabidopsis*orthologuegene model(residue)	Amino acididentity^b^(%/residue)
*bTUB*	β-Tubulin	721/727/730	221	3	RGCCCTCTAYGATATYTGCTTCCGYAC(A L D Y I C F R T)	beta-Tubulin#1beta-Tubulin#2beta-Tubulin#3	AB824713AB824714AB824715	*At1g75780*(447)	91.7/241
*HIS*	Histone H3	288	337	2	TACCGCCCTGGTACTGTYGCTCTTC(Y R P G T V A L )	HistoneH3#1HistoneH3#2	AB824718AB824719	*At5g10980*(136)	100/95
*EF1a*	EF-1α	627	331	2	GATGTGTACAAGATTGGTG(D V Y K I G )	EF-1alpha#1EF-1alpha#2	AB824716AB824717	*At5g60390*(449)	92.9/210
*ANX*	Annexin	933	281	2	CCAGATGTGGTTCCTTCTCCAGTG(P D V V P S P V )	Annexin#1Annexin#2	NotregisteredAB826126	*At5g10220*(318)	67.1/310
*ACT*	Actin	1063	491	2	TGGTGATGATGCTCCCAGGGCTGT^c^(G D D A P R A V)	Actin#1Actin#2	AB826124AB826125	*At5g09810*(377)	98.9/353
*GAP*	GAPDH	1026	469	2	ATCATGGCAACATCTGGCAAGAAG(M A T S G K K )	GAPDH#1GAPDH#2	AB826122AB826123	*At1g13440*(338)	89.7/341
*SAND*	SAND protein	951	231	1	TCYCTCATCCAYTCWTTCAGTTG^c^(P S S T H S V )	SAND#1	AB795982	*At2g28390*(607)	72.2/316
*TIP*	TIP41 protein	797	223	1	GYATCCATGAYTGGGARATCGA^c^(I H D W E I E)	TIP41#1	AB795983	*At4g34270*(290)	68.6/264
*CBF2*	CBF2	382	91		TAGAGGGARGCTTGCCTGCCTCAA	CBF#1412	AB826494	*At4g25470*	44.4/126
*CBF4*	CBF4	433	151		(R G R/K L A C L N)	CBF#1403	AB826495	(216)	42.0/143

a Nucleotide numbers in the longest clone.

b Highest value among 3′-RACE clones to *Arabidopsis* orthologues.

c Nested primer used for secondary amplification is shown.

### Expression Levels of Reference Genes

To explore gene expression levels within homologous genes, we designed 18 combinations of primer sets for RT-qPCR. Although we obtained two annexin RACE clones, a primer pair corresponding to *Annexin#1* clone did not amplify any fragment. Therefore, we tested the remaining 17 primer sets (Table S4) for reference gene evaluation. Three primer sets (*HIS*_cds, *EF1a*_cds and *ANX*_cds) amplified within coding sequence; while all others amplified sequences across stop codons, which means the amplified fragment contains CDS and flanking 3′-UTR. All the primer sets amplified single fragments as confirmed by melting curve analysis (data not shown) with Tm values ranging from 79 to 85°C (Table S4); and also confirmed by direct sequencing of amplified fragments (Figure S1A to S1Q). Efficiency of amplification was 91–112% (Table S4). Elimination of possible contaminations of genomic DNA in RNA samples was confirmed by performing reverse transcriptase-omitted (–RT) negative control experiment (Figure S2) using *SAND*_utr primers. No amplified fragments were observed for –RT samples, while a. 1.2 kb fragment was obtained when genomic DNA was used as template. From the sequencing result of genomic fragment by *SAND*_utr (Figure S1P), *SAND* gene has an 1155 bp-long intron between the primer sites.

**Figure 1:**
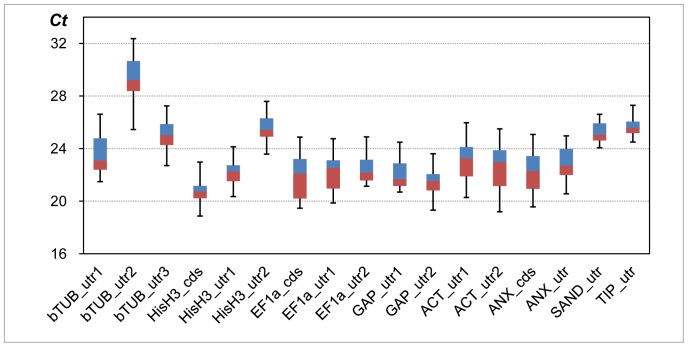
Distribution of threshold cycle (*Ct*) of tested reference gene regions. Lower line, lower box, upper box and upper line indicate percentiles of up to 25, 50, 75 and 100% range, respectively.

Distribution of threshold cycle number (*Ct*) values for each primer regions are shown in Figure 1. *HIS*_cds was most abundantly expressed (median of *Ct* [m*Ct*], ca. 21); *GAP*_utr2 (m*Ct*, ca. 22) was the second. m*Ct* for the newly added gene regions *ANX*_cds and *ANX*_utr were ca. 23, while that of *SAND*_utr and *TIP*_utr were ca. 25 and 26, respectively, indicating expression levels of these new genes were 1/4 to 1/30 of the most abundant gene (*HIS*_cds) in our Japanese pear samples. The most weakly expressed gene region was *bTUB*_utr2 with m*Ct* ca. 29. The expression level of this gene was estimated to be ca. 1/300 relative to the *HIS*_cds.

### Comparison of Template Amount between CDS and 3′-UTR

Differences in *Ct* values between the CDS and 3′-UTR regions in the same gene were mostly within one cycle in the 3 genes tested: *HIS*, *EF1a* and *ANX* (data not shown). When the ratio of initial template amount was calculated as described in Materials and Methods, more templates existed in CDS than 3′-UTR in *HIS* and *EF1a* (Table 2). This suggested that the CDS primer pair can amplify multi locus homologous genes, while the 3′-UTR primer can amplify single locus gene. For *ANX*, the ratio ranged from 0.2 to 0.7, suggesting amplification from single locus. The flower bud (FB) group exhibited the lowest values in all three genes, suggesting much fewer CDS templates than in other groups.

**Table 2:** Ratio (mean and standard deviation) of initial amount of cDNA templates between CDS and 3′-UTR primer regions in the same gene.

Gene_region	Total	FB	FO	FF	FS
*His*_cds/utr1	1.222±0.459	0.609±0.017	1.368±0.112	1.660±0.467	1.133±0.117
*EF1a*_cds/utr1	1.075±0.243	0.718±0.087	1.112±0.084	1.274±0.153	1.276±0.036
*ANX*_cds/utr	0.506±0.216	0.200±0.026	0.575±0.152	0.628±0.117	0.668±0.172

Initial amount of cDNA template was calculated from *Ct* value and efficiency of PCR as described in the Materials and Methods. Sample group symbols are as in Table 6.

### Gene Expression Stability of Reference Genes

**Figure 2:**
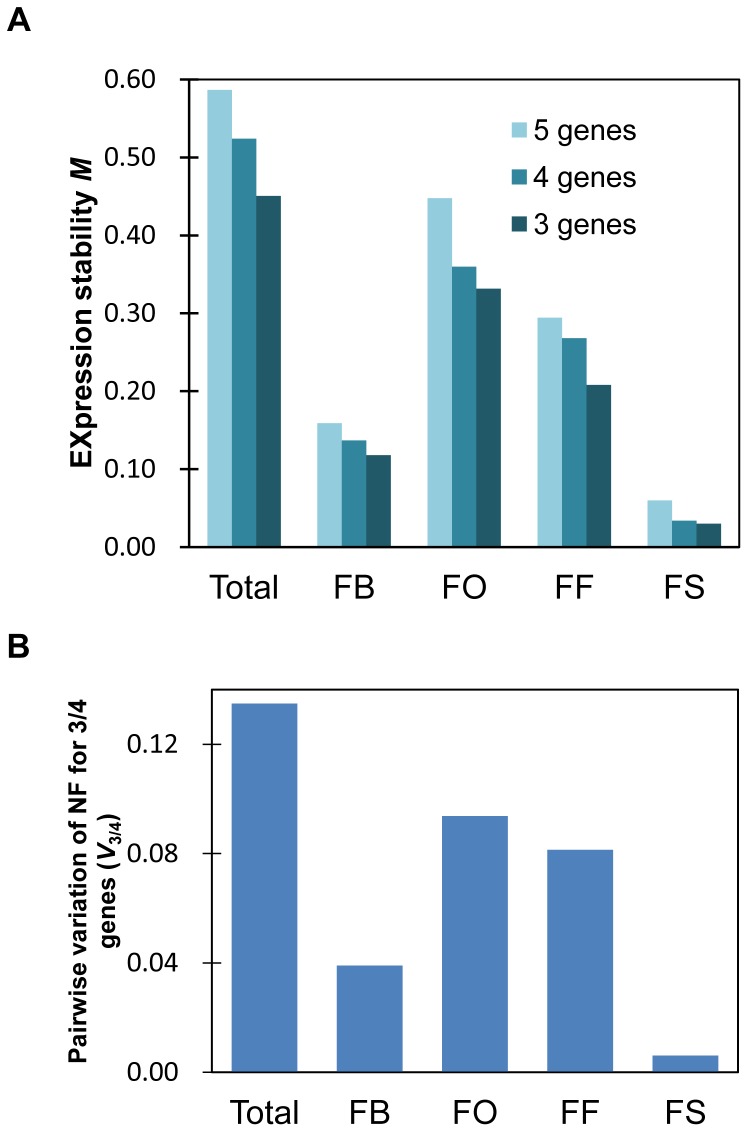
Parameters calculated from geNorm analysis. (A) Average expression stability value *M* for best performed 5, 4 or 3 genes. (B) Pair-wise variation of normalization factors for best performing 3 or 4 genes (*V*_3/4_). Group symbols are as in Table 6.

The geNorm algorithm [2] is widely used for gene expression stability analysis. When our data were processed by geNorm, best and worst gene regions were as shown in Table 3, and Figure 2A. For FB samples, data obtained by using CDS primers (*HIS*_cds, *EF1a*_cds and *ANX*_cds) were omitted from ranking because of the different primers employed for reverse transcription (see Materials and Methods). The best three genes are different among sample groups, while *EF1a*_cds and *EF1a*_utr1 were commonly ranked in flower organ (FO) samples, fruit flesh (FF) and fruit skin (FS). For overall samples, *HIS*_cds, *SAND*_utr and *TIP*_utr were the three top-ranked genes although these genes were not ranked as the best three in each subgroup. For FO, stability values are relatively larger than other groups indicating a less stable expression than other groups, probably reflecting variation of gene expression in each flower organ. Pair-wise variation of normalization factor for 3 or 4 genes (*V*_3/4_) were calculated as shown in Figure 2B. *V*_3/4_ value was less than 0.1 for each sample group, 0.135 for overall samples.

**Table 3:** Stability ranking of reference gene regions evaluated by geNorm.

Rank	Total		FB		FO		FF		FS	
1–3	*HIS*_cds	0.450	*bTUB*_utr3	0.118	*EF1a*_cds	0.331	*EF1a*_cds	0.208	*HIS*_utr1	0.031
	*SAND*_utr		*HIS*_utr2		*EF1a*_utr1		*EF1a*_utr1		*EF1a*_cds	
	*TIP*_utr		*ANX*_utr		*ACT*_utr1		*TIP*_utr		*EF1a*_utr1	
4	*HIS*_utr1	0.524	*HIS*_utr1	0.137	*ACT*_utr2	0.360	*GAP*_utr2	0.268	*TIP*_utr	0.034
5	*EF1a*_utr2	0.586	*SAND*_utr	0.159	*ANX*_cds	0.448	*bTUB*_utr1	0.294	*EF1a*_utr2	0.060
15	*bTUB*_utr3	1.094	*ACT*_utr2	0.356*	*bTUB_*utr1	0.720	*ANX*_utr	0.822	*HIS*_utr2	0.798
16	*ACT*_utr2	1.136	*GAP*_utr2	0.425*	*bTUB_*utr2	0.821	*ANX*_cds	0.901	*ANX*_cds	0.885
17	*bTUB*_utr2	1.236	*bTUB*_utr2	0.536*	*bTUB_*utr3	0.934	*bTUB*_utr2	1.115	*bTUB*_utr2	1.047

Average expression stability value *M* was calculated for all 17 gene regions and least stable gene region (with highest *M* value) indicated in the left column was excluded and this stepwise exclusion was repeated until three genes remained. Top five and last three gene regions are indicated. Sample group symbols are as in Table 6. For FB samples, *M* was calculated without data obtained by CDS primers. Accordingly, ranking of least stable genes indicated by asterisks were 12 to 14, respectively.

**Figure 3:**
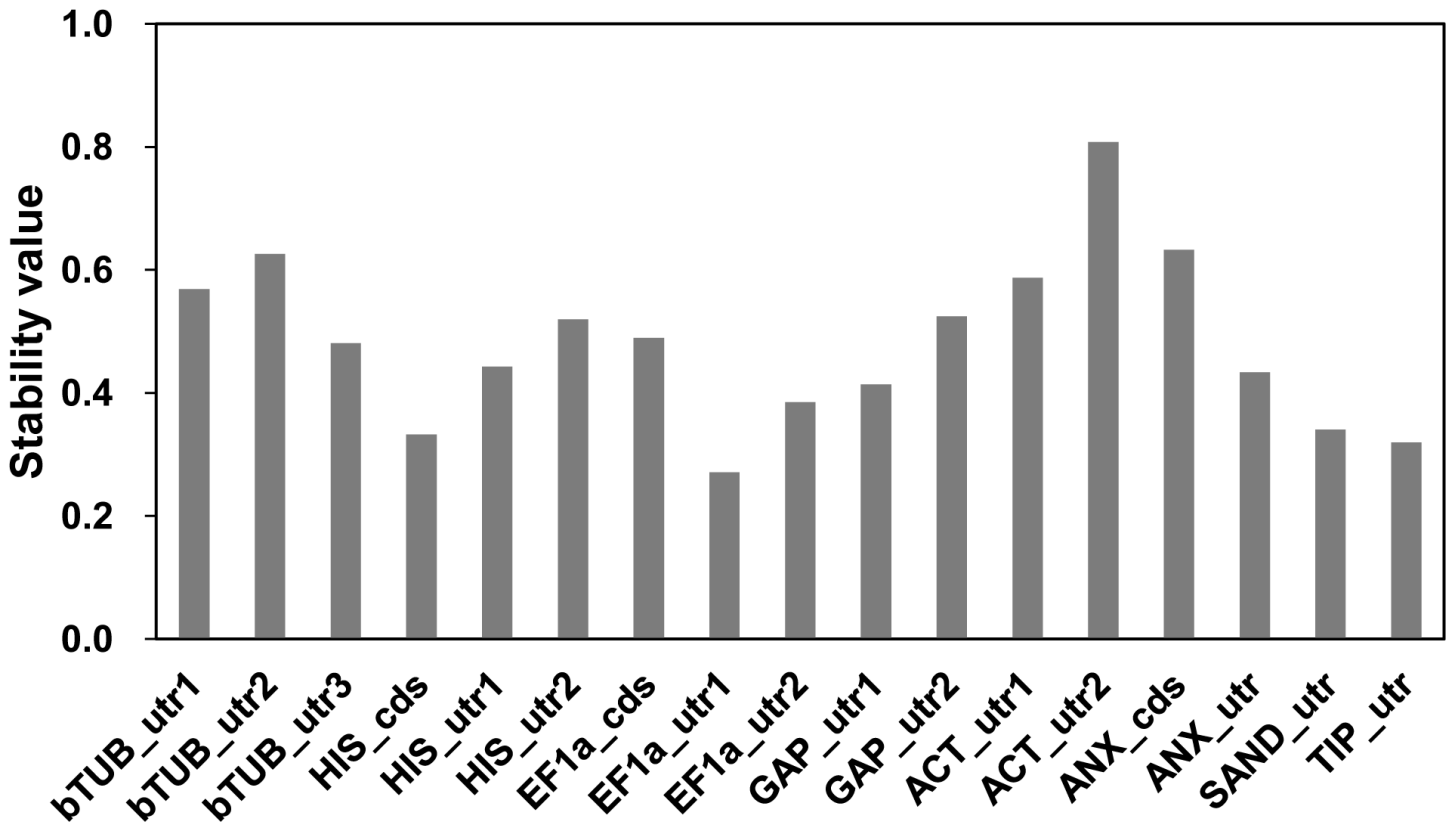
Stability value calculated from NormFinder.

Gene expression stability evaluated by NormFinder [16] is shown in Figure 3 (for overall samples) and Table 4 (for each sample group). The best gene region was *EF1a*_utr1 (stability value 0.271) and best combination for two genes was *EF1a*_utr1 and *EF1a*_utr2 (0.101). Although intra-group variations were different in each sample group, stability values were relatively small in the best performed gene regions in each sample group (Table 4), suggesting that some genes were expressed quite stably with respect to sample group.

**Table 4:** Stability ranking of reference gene regions evaluated by NormFinder.

Gene_region	FB	FO	FF	FS
*bTUB*_utr1	0.054	0.353	***0.002***	0.170
*bTUB*_utr2	0.551	0.736	1.192	1.642
*bTUB*_utr3	0.018	1.322	0.573	0.467
*HIS*_cds	0.042	0.057	0.139	0.036
*HIS*_utr1	0.021	0.087	0.283	***0.000***
*HIS*_utr2	***0.006***	0.104	0.314	0.706
*EF1a*_cds	0.100	***0.049***	0.016	***0.002***
*EF1a*_utr1	0.045	0.074	***0.006***	***0.000***
*EF1a*_utr2	0.100	0.174	0.049	0.006
*GAP*_utr1	0.030	0.124	0.048	0.260
*GAP*_utr2	0.338	0.200	0.032	0.005
*ACT*_utr1	0.025	***0.015***	0.280	0.287
*ACT*_utr2	0.080	0.052	0.451	0.339
*ANX*_cds	***0.015***	0.308	0.737	0.575
*ANX*_utr2	0.036	0.276	0.530	0.257
*SAND*_utr	0.058	***0.014***	0.051	0.044
*TIP*_utr	***0.014***	0.080	***0.007***	0.002

Intra-group variation of stability value calculated from NormFinder. Sample group symbols are as in Table 6. Top three-ranked gene regions are in bold and italic.

ΔCt method evaluates gene expression stability by calculating pair-wise differences of *Ct* (ΔCt) and ranked by standard deviation of ΔCt [18]. The results obtained are shown in Table 5. For the overall samples, the result was similar to geNorm analysis: top three-ranked gene regions, namely *SAND*_utr, *TIP*_utr and *HIS*_cds were the same. For each sample group, the top three-ranked gene regions were somewhat different from geNorm results. Two of three were common (*bTUB*_utr3 and *ANX*_utr) in FB; while only one gene was commonly found, *ACT*_utr1 in FO, *TIP*_utr in FF and none in FS (Table 3 and 5). *SAND*_utr ranked as the best three gene regions in all sample groups, suggesting standard deviation of ΔCt with *SAND*_utr was relatively small.

**Table 5:** Stability ranking of reference gene regions evaluated by ΔCt.

Rank	Total		FB		FO		FF		FS	
1	*SAND*_utr	0.798	*ANX*_utr	0.377	*SAND*_utr	0.689	*TIP*_utr	0.658	*HIS*_cds	0.485
2	*TIP*_utr	0.829	*SAND*_utr	0.392	*HIS*_cds	0.751	*SAND*_utr	0.689	*SAND*_utr	0.491
3	*HIS*_cds	0.883	*bTUB*_utr3	0.393	*ACT*_utr1	0.779	*EF1a*_utr2	0.713	*EF1a*_utr2	0.532
4	*EF1a*_utr2	0.947	*EF1a*_utr2	0.444	*TIP*_utr	0.786	*GAP*_utr2	0.721	*GAP*_utr2	0.555

Gene expression stability ranking by ΔCt method. Eight gene regions were selected based on the narrow range distribution of *Ct* values; one each from different homologous gene groups. Average values of standard deviation of ΔCt for all pair-wise combinations were calculated. Sample group symbols are as in Table 6.

### Gene Expression of *PpCBF2* and *PpCBF4*

**Figure 4:**
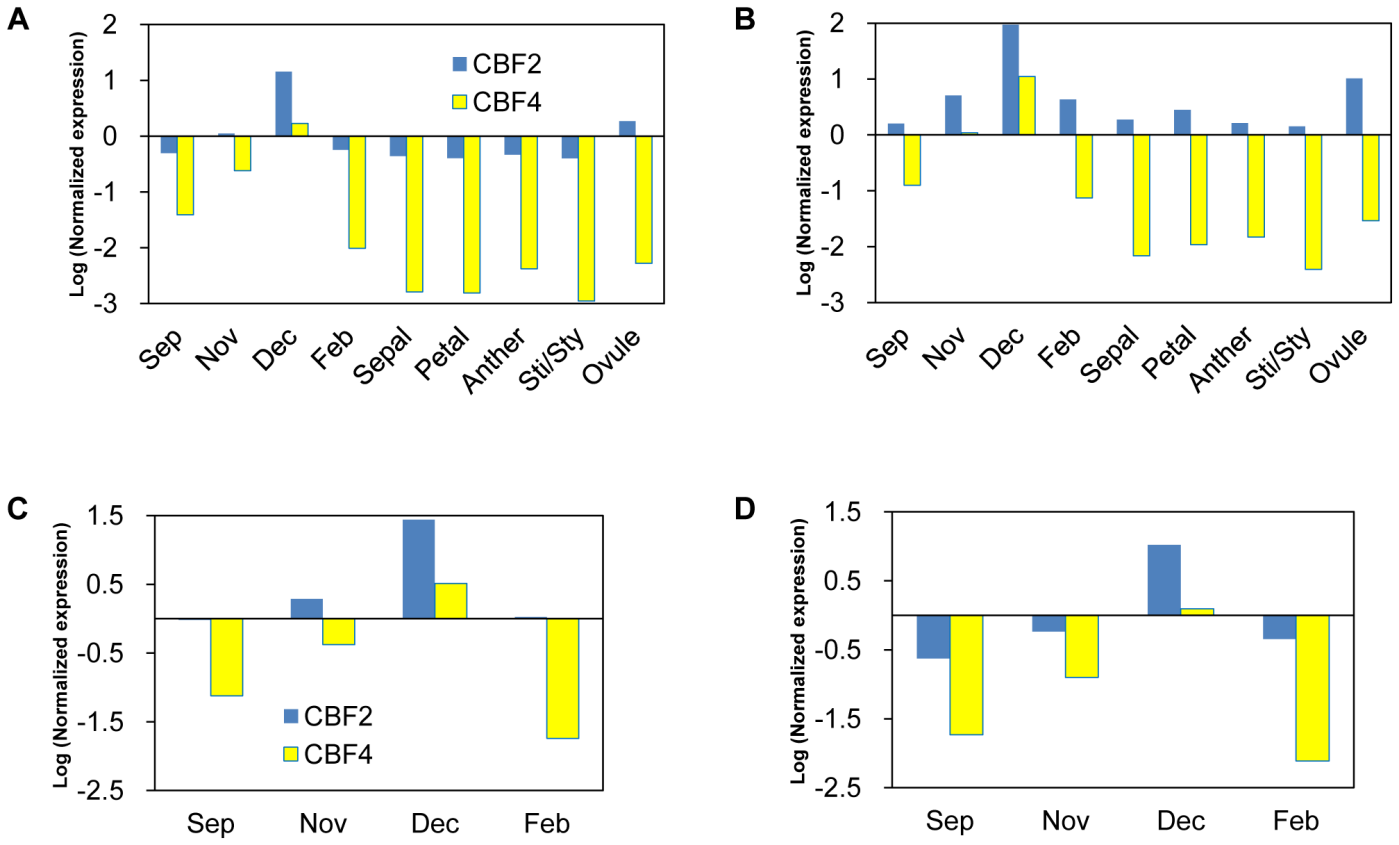
Normalized expression level of *PpCBF2* and *PpCBF4*. (A) Normalization by best stable genes for overall samples (*HIS*_cds, *SAND*_utr, *TIP*_utr). (B) By least stable genes for overall samples (*bTUB*_utr3, *ACT*_utr2, *bTUB*_utr2). (C) By best stable genes for FB (*bTUB*_utr3, *HIS*_utr2, *ANX*_utr). (D) By least stable genes for FB (*EF1a*_cds, *GAP*_utr2, *bTUB*_utr2).

By using ranking of gene expression stability evaluated by geNorm, the best and worst three gene regions were selected (Table 3) for normalization of *PpCBF2* or *PpCBF4*. A normalization factor using three genes (NF_3_) was defined as geometric mean of relative expression value in the three gene regions in each sample [2]. The expression levels of these cold-inducible genes were quite low in FF and FS samples (data not shown). Accordingly, normalized expression levels in FB and FO samples are shown in Figure 4A (by three best genes) and B (by three worst genes). Expression level was remarkably larger in *PpCBF2* than in *PpCBF4* with similar seasonal fluctuations which peaked in December. Due to the difference of calculated NF_3_, expression level was 3- to 8-fold larger when normalized by the least three stable genes. However, within each sample group, expression levels were quite similar in both cases. This is also the case when using the best and worst three gene regions suggested within FB samples as shown in Figure 4C and D, respectively.

## Discussion

We have evaluated five widely used reference genes in RT-qPCR studies in plant research together with newly added three genes suggested by robust expression from microarray studies in *Arabidopsis* using RNA samples from Japanese pear. Several different cDNA sequences were cloned from 3′-RACE fragment as shown in Table 1. Deduced amino acid sequences were compared with orthologue sequences in *Arabidopsis*. The identity was roughly more than 90% for the five traditional genes *bTUB*, *HIS*, *EF1a*, *GAP* and *ACT* (Table 1). For the new three genes tested (*SAND*, *TIP* and *ANX*), amino acid identities were relatively low (about 70%). Similar identities were reported in *Eucalyptus* or tomato orthologous genes [9], [22].

To evaluate gene expression by RT-qPCR, primer sets were designed across stop codons (Table S4) which enabled gene-specific amplification. For comparison, CDS primer sets were also included for 3′-RACE clones such as *HistoneH3#1*, *EF-1alpha#1* and *Annexin#2*, and the ratio of initial amount of templates having CDS region versus 3′-UTR region was evaluated. Distributions of *Ct* values (as summarized in Figure 1) indicated that most of the gene regions tested were with *Ct* values between 20 and 24. *Ct* values of the newly added *TIP*_utr and *SAND*_utr were relatively higher by two to five cycles compared to *ACT* or *GAP* gene regions traditionally used. Similar results were also reported in *Eucalyptus*, papaya, grapevine, hop and banana [9], [21], [33], [34], [37]. For *bTUB* or *HIS* gene homologue, *Ct* values fluctuated among genes suggesting different levels of expression within homologous genes (Figure 1). For example, difference of m*Ct* was six between *bTUB*_utr1 and _utr2, suggesting a 30- to 100-fold difference of gene expression between the two genes. Differences of expression levels within homologous genes were also reported in *UBI* or *bTUB* genes in *Arabidopsis* [12]. On the other hand, *EF1a*, *GAP* and *ACT*, gene expression levels were similar between gene homologues (_utr1 and _utr2 regions). Ratio of initial cDNA amount corresponding to CDS versus 3′-UTR regions were calculated as shown in Table 2. For *HIS* and *EF1a* gene regions, more cDNA templates existed in CDS than 3′-UTR, except for FB samples. The results suggested that multiple gene homologues can be amplified with the tested CDS primer sets. Direct sequencing analysis of amplified fragment with *EF1a*_cds or *HIS*_cds primers revealed that almost no polymorphic sequences in the region (Figure S1D and S1G). From these results, it seems that multiple gene homologues having the same CDS sequences from different gene loci with different 3′-UTR sequences are existed. To clarify this, sequence analysis of many 3′-RACE clones or RNA-Seq is needed. If above case is true, *Ct* values obtained by CDS primers indicated overall gene expression in several homologous gene regions. This may be convenient for gene expression studies across highly divergent samples because we can monitor gene expression levels much widely. Indeed, according to geNorm analysis, *His*_cds was good performing with overall samples, and *EF1a*_cds also ranked well in FO, FF and FS samples (Table 3). Thus, for genes existing as multiple homologues, gene expression levels are often diverse for individual homologue and care should be taken for primer setting in CDS. Evaluation of initial template amount between *ANX*_cds and *ANX*_utr regions by a calculated ratio were 0.58 to 0.67 in the FO, FF and FS samples. The ratio was extraordinarily low with a value of 0.20 in FB samples (Table 2). For *HIS* and *EF1a* gene regions, a low value tendency was similar in FB samples. We suppose the primary reason for this result is due to the differences in the primers used for reverse transcription: FB cDNAs were synthesized with oligo-dT primer, whereas FO, FF and FS templates were with random primers. Based on the results obtained, when using oligo-dT primer, amount of cDNA templates with more than 0.5 kb-long seems to be roughly half of that with about 0.3 kb-long. In *Arabidopsis*, Czechowski et al. [6] reported that the initial template ratio of 5′- and 3′-primer regions occurred about 0.95 kb distance in *GAP*, mostly ranging from 0.5 to 0.7, while several sample groups exhibited below 0.4, basically similar to our current studies. Taken together, if RT-qPCR primers amplifying CDS regions were to be used, care should be taken again on the integrity of RNA and subsequent synthesized cDNA samples especially using source materials of diverse origin for comparison.

geNorm algorithm evaluates gene expression stability based on log_2_-transformed expression ratios for all pair-wise gene combinations and calculates their standard deviation (*V* value). Step-wise elimination of a gene exhibiting the highest *V* value and recalculation of *V* was repeated until three genes remained. The average *V* for a certain gene is an indicator of stability expression value *M* [2]. In the present study, *M* value for best performed three genes on the overall samples were 0.450 (Figure 2A and Table 3) and from 0.031 to 0.331 for individual sample groups. Czechowski et al. [6] reported *M* value for most stably expressed genes of about 0.6 for microarray data set, and 0.7 for subsequently selected genes evaluated by RT-qPCR. Therefore, three best performed genes in the current study shown in Table 3 are well-suited for normalization purposes. Vandesompele et al. [2] also proposed a method to determine the number of the genes needed for normalization by pair-wise variation analysis between the normalization factors (NF) NF*_n_* and NF*_n_*_+1_ with the same calculation formula applied for quantification of gene expression as described. Based on the data obtained, a cut-off value of 0.15 for *V_n_*_/*n*+1_ was proposed [2]. In our present study, *V*_3/4_ is lower than this cut-off value (Figure 2B), suggesting three best genes are enough for normalization.

NormFinder algorithm is a model-based approach for estimation of expression variation [16] that takes into account intra- and inter-group variations for normalization factor calculation. Stability values calculated were shown in Figure 3, ranging from 0.271 (*EF1a*_utr1) to 0.808 (*ACT*_utr2) for overall samples. Best combination of two genes suggested is *EF1a*_utr1 and *EF1a*_utr2 with a stability value of 0.101. This recommendation is different from geNorm, probably due to compensation effect of expression levels with these two genes that is taken into account with NormFider. For evaluation of intra-group variation, stability values calculated in each group were compared (Table 4). The best performed three gene regions (indicated in bold italic in Table 4) were basically similar to the five top-ranked gene regions as shown in Table 3. Although the calculation method is different, similar results of gene expression stability between geNorm and Normfinder strengthened the validity of evaluation of these methods.

ΔCt method is a relatively simple approach to calculate stability based on ΔCt for all pair-wise gene combinations. The results shown in Table 5 were somewhat different from geNorm or NormFinder, probably due to using only *Ct* value for the calculation. In practice, efficiency of PCR fluctuates along with sequences of primers or amplification targets, coexisting substances originated from RNA extract. These factors affecting quantification of the target sequences are not considered in ΔCt method. However, simplicity of the method makes it still largely advantageous to use. To evaluate validity of the method, coefficient of variance (CV) was calculated for results shown in Table 5. CV for the three best genes is 0.051 for overall samples, 0.023 to 0.062 for individual sample groups. These CV values seem to be sufficient for practical use. ΔCt method can be applied without making standard curve. Consequently, fewer samples are needed. For this useful feature, ΔCt method will be suitable for initial screening of reference genes from ten or more candidates. Then five or six genes are selected, and quantification using standard curve and subsequent evaluation by geNorm or NormFinder is applied to find out the best performing three or four reference genes in the samples of interest. This may be a practical approach to find out appropriate reference gene combinations with time- and labor-saving considerations.

To compare normalization results practically, the three best and worst ranked gene regions in Table 3 were used to calculate NF and subsequent quantification of *PpCBF2* or *PpCBF4* expressions. Expression of both genes transiently increased under cold environment, after peaking in December, gene expression rapidly reduced. This pattern resembles that of dormancy-associated MADS-box genes in Japanese pear [26] or a dehydrin gene and also its protein product in Japanese apricot [38]. There were remarkable increases in expression levels when normalization was done by the best genes for FB, namely *bTUB*_utr3, *HIS*_utr2 and *ANX*_utr, compared to normalization by the best genes for overall sample, *HIS*_cds, *SAND*_utr and *TIP*_utr (Figure 4A and C). Comparing the results using normalization with the most and the least stable three reference genes, 3- to 8-fold larger values were quantified by using least stable reference genes. On the other hand, relative expression values were unexpectedly basically similar within sample group (FB or FO) in both results (Figure 4A and B). This suggested robustness in quantification when three genes were used to calculate NF in each sample. Using multiple reference genes seems especially important for testing experimental samples derived from various sources as Vandesompele et al. [2] and Løvdal and Lillo [15] suggested. Although many reports are being published regarding the evaluation of reference genes in various plant species, tissue types or treatments, normalization by using multiple reference genes seems not popular yet in plant studies and should be used widely in future.

Conclusively, we have confirmed superiority of using multiple reference genes for RT-qPCR data using various samples from Japanese pear. To find out suitable reference genes practically, we propose utilization of ΔCt method as a first approach to testing stability before subsequent evaluation by geNorm or NormFinder.

## Materials and Methods

### Plant Materials

We used different tissue samples collected from 36-year old field-grown trees of the Japanese pear (*P. pyrifolia*) cv. ‘Kosui’ grown at the orchard of the NARO Institute of Fruit Tree Science, Tsukuba, Japan (lat. 36°N, long. 140°E). A descriptive list of the various tissue samples is provided in Table 6. For the gene expression analysis in the flower organs, several flowers were collected (1-day prior to flower opening) in April 2011 and separated into the different flower parts (including also whole flower sample). To investigate the variation in gene expression during cold stress, the flower bud samples were collected at intervals during the 2010/2011 seasonal cold transitions [autumn–winter]. The date of sampling [temperatures of seasonal average (1981–2010); min. – max. of the sampling day] were as follows: Sep. 30 (19.0°C; 16.8–19.0°C), Nov. 18 (9.4°C; 4.2–13.8°C), Dec. 15 (4.9°C; 3.2–12.9°C) and Feb. 16 (3.9°C; –4.9–10.1°C). Temperature data were recorded at “Tateno” measuring point of Japan Meteorological Agency, located about 2 km eastward from our experimental field. Sprouting ratio of ‘Kosui’ pear leaf bud under an ambient condition on these dates were roughly less than 10%, 0%, 50% and 100%, respectively [26]. Because cutting inherently influences endodormancy condition, it is difficult to measure sprouting ratio with similar treatment using flower buds on cut branches (data not shown). Furthermore, fruit tissue samples (cortex and skin) were collected at different developmental stages in order to investigate the variation in gene expression during these developmental phases. The commercial harvesting date at full maturity was Aug. 21. Flower organs were separated from balloon stage flowers with forceps. All the collected samples were immediately frozen in liquid nitrogen and stored at –80°C until needed for RNA isolation.

**Table 6:** A descriptive list of the different samples used in this study.

Group	Tissue type	Number ofsamples	Dates/Seasonscollected	Experimental significance
**FB**	Flower bud	4	Sep. 30, Nov. 18Dec. 15, Feb. 16	Hardening progression/endodormancy development and release (2010–2011)
**FO**	Flower organs	6	April (1 day prior toflower opening)	Sepals, petals, anthers, stigmas+styles and ovules manually separated with forceps and whole flowers
**FF**	Fruit flesh	4	May 22, Jul. 3,Aug. 12, Aug. 25	Early, middle, late and postharvest developmental stages (2009) Commercial harvest day: 21 Aug.
**FS**	Fruit skin	2	Jul 3, Sep. 3	Middle and postharvest developmental stages (2009) as above

### Total RNA Isolation and First Stand cDNA Synthesis

Total RNA was isolated from ‘Kosui’ flower bud, flower and fruit samples collected as shown in Table 4 using hot borate extraction procedure [39]. The synthesis of the first-strand cDNA was made using the SuperScript® VILO™ First Strand cDNA Synthesis System (Invitrogen, Life Technologies, Tokyo, Japan). The 2.0 µg aliquot of total RNA used in the reaction was first treated with RQ1 DNase (Promega, Madison, WI) and was reverse-transcribed using random hexamer primers or oligo-dT primers (for FB only) according to the manufacturer’s instructions. The cDNAs were diluted to a final concentration of 10 ng/µl with sterile MilliQ water prior to RT-qPCR analysis. To confirm complete degradation of genomic DNA in RQ1 DNase-treated RNA samples, RT-qPCR primer pairs for *SAND* were also used for reverse-transcriptase-less (–RT) controls. Two representative samples from each FB, FO, FF and FS sample groups (Table 6) were subjected to ordinary PCR. The mixture contained 200 µM dNTPs, 0.1 µM *SAND*_utr primer pairs (Table S4), 0.6 units of *Ex Taq* DNA polymerase (TaKaRa Bio) and 0.8 µl (5–10 ng equivalent) cDNA in total volume of 15 µl. PCR was performed using the following thermal cycling conditions: 95°C for 60 s, 42 cycles of (94°C for 20 s, 60°C for 30 s, 72°C for 30 s), and 72°C for 150 s.

### Selection of Candidate Reference Genes, Cloning and Annotation of Gene Fragments

Based on previous reports evaluating stability of gene expression in woody plants, eight candidate genes were selected for this study to investigate their robustness as reference genes for RT-qPCR in *P. pyrifolia*. Special consideration was given to ensure the selection of genes belonging to different functional classes (Table S1) in order to significantly reduce the probability of using co-regulated genes. In addition to most frequently used genes such as *bTUB*, *ACT*, *HIS*, *EF1a* and *GAP*, three new genes were added: *SAND*, *TIP* and *ANX*. The new genes were selected originally based on the results showing relatively stable gene expression pattern on microarray experiments in *Arabidopsis* [6]. The eight candidate reference genes used in this study and the primers for the cloning of the gene fragments using 3′- RACE are shown in Table 1. The RACE primers were selected from the conserved nucleotide region corresponding to genomic or EST sequences of the respective genes in apple or grapevine. A 2.5 µg aliquot of total RNA isolated from Japanese pear ‘Kosui’ flower buds collected in the winter season was used to prepare 3′-RACE-Ready cDNAs with a SMART RACE cDNA Amplification Kit (Clontech, TaKaRa Bio, Shiga, Japan) according to the manufacturer’s instructions. RACE fragments were amplified using 1.0 µl of this cDNA with ‘touchdown’ PCR program: 94°C for 45 s, 4 cycles of (94°C for 20 s, 66°C for 25 s, 72°C for 75 s), 4 cycles of (94°C for 20 s, 64°C for 25 s, 72°C for 75 s), 38 cycles of (94°C for 20 s, 62°C for 25 s, 72°C for 75 s), and 72°C for 150 s. Where clear amplified fragment was not obtained, first PCR product was one-fiftieth diluted, then nested PCR with a second primer was performed using the following program: 94°C for 45 s, 6 cycles of (94°C for 20 s, 66°C for 25 s, 72°C for 75 s), 26 cycles of (94°C for 20 s, 63°C for 25 s, 72°C for 75 s) and 72°C for 300 s. The amplified fragments were cloned into the pCR2.1-TOPO vector using TOPO TA cloning Kit (Invitrogen) and sequences were analyzed by the 3130xl Genetic analyzer (Applied Biosystems, Life Technologies), and annotated to confirm each gene identity. Based on the sequences obtained, the number of homologous genes was estimated and primer sets for RT-qPCR for each reference gene homolog (Table S4) was selected by Primer 3 software ver 0.4.0 (http://frodo.wi.mit.edu/; [40]). In addition, two *P. pyrifolia* cold-inducible target genes, namely *PpCBF2* and *PpCBF4* were included to illustrate the robustness of the reference genes. The cold-inducible genes were also cloned from the 3′-RACE fragment (as indicated above) using the gene specific primer, 5′-TAGAGGGARGCTTGCCTGCCTCAA-3′, applicable to both genes and the gene numbers were designated based on the apple *CBF* genes (*MdCBF2* found in contig MDC013783.459 and *MdCBF4* as predicted ORF MDP0000031450 in MDC027186.43, [41], [42]). The specific RT-qPCR primers were then designed from these fragments as indicated above for the reference genes. The designations of the gene fragments used in this study and their DNA Data Bank of Japan (DDBJ) accession numbers are as shown in Table 1.

The symbols of the genes, full gene names and the region of the amplicons analyzed by RT-qPCR (as shown in Table S4) are as follows: *bTUB* (*β-Tubulin*), 3′-untranslated region [_utr]1, 2 or 3; *HIS* (*Histone H3*), coding sequence [_cds], _utr1 or 2; *EF1a* (*Elongation Factor-1α*), _cds, _utr1 or 2; *ANX* (*Annexin*), _cds or _utr; *GAP* (*Glyceraldehyde-3-phosphate dehydrogenase*), _utr1 or 2; *TIP* (*TIP41-like protein*), _utr; *SAND* (*SAND family protein*), _utr; *ACT* (*Actin* ), _utr1 or 2. The specific RT-qPCR primers for cold-inducible target gene of interest *CBF2* (*C-repeat binding factor 2*) _utr and *CBF4*_utr are also shown in Table S4.

### RT-qPCR

We tested 17 regions of candidate reference genes probably representing 15 loci, together with two regions of target gene of interest (i.e. cold-inducible gene) as shown in Table S4. Prior to RT-qPCR, the specificity of the primer set for each gene was first tested by electrophoresis of amplified products on 1.5% high-grade agarose gel in which single products were observed (data not shown). In addition, direct sequencing of amplicons was performed to confirm validity of primers for RT-qPCR (Figure S1). Amplicon fragments were generated from FB cDNA sample on Dec. 15 using RT-qPCR primer pairs listed in Table S4. Approximately, 2 µL aliquot of the amplified fragments was directly sequenced by either one of the respective primer. To confirm entire sequence of amplicons, sequencing of both directions was done. The real-time quantification of the first strand cDNA was performed on the ABI 7500 Real Time PCR System (Applied Biosystems) and analyzed with the ABI 7500 Software ver.2.0.4. The reaction mixture (10 µl) contained 1.0 µl of cDNA sample (equivalent to 10 ng of the initial total RNA), 0.4 µM of each primer, and 5 µl of SYBR® Premix *ExTaq* II (2×) (TaKaRa Bio) and 0.2 µl of Rox Reference Dye II (TaKaRa Bio). For a negative control reaction, no template was added to the reaction mixture, which resulted in no detectable fluorescence signal from the reaction. RT-qPCR conditions were set as follows: initial denaturation for 30 s at 95°C, followed by 40 cycles of denaturation at 95°C for 5 s, and annealing and extension for 34 s at 60°C. After amplification cycles, each reaction was subjected to melting-temperature analysis to confirm single amplified products: 95°C for 15 s, 60°C for 60 s, 1% slope elevation until 95°C and held for 30 s, followed by final step at 60°C for 15 s. The fluorescence decreased at a single discrete temperature indicating the separation of both strands of a single DNA species. Plasmid DNA solutions containing 3′-RACE cDNA fragment was used as standard samples for making standard curves for quantification over four cycles of one tenth dilution with 10 mM Tris-HCl (pH 8.0). Relative quantities of these standard samples were thus set as 100, 10, 1, 0.1 and 0.01, and quantities of tested cDNA samples (listed in Table 4) were calculated by the ABI 7500 Real-Time PCR System software ver.2.0.4. Three replications (wells) were used for each sample. This experiment was repeated twice in independent analytical runs for all the genes (reference gene and target gene of interest). The *Ct*, Tm of amplified fragment and PCR efficiency for each reaction was automatically determined by the System software ver.2.0.4 with default parameters. For the included two cold-inducible genes, *PpCBF2* and *PpCBF4*, their transcript levels were normalized against the transcript levels of best performed three reference genes or least three genes to establish the relative expression value for comparison.

### Comparing Amounts of cDNA Templates Using CDS Primers versus 3′-UTR Primers

For quantification of CDS versus 3′-UTR fragments originated from the same gene, a threshold value of baseline-subtracted fluorescence signal (*Rn*) was set at 0.3, and the ratio of initial templates was calculated [6]:
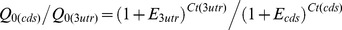
where *Q_0_* is initial amount of template and *E* is efficiency of PCR.

### Statistical Analysis of Gene Expression Stability

For evaluating gene expression stabilities, three different methods were used: geNorm [2], NormFinder [16] and ΔCt [18]. *Ct* values from RT-qPCR experiment were converted into relative quantities according to standard curve obtained in each PCR run and the resultant quantities were processed by geNorm or NormFinder. ΔCt method is relatively simple because *Ct* values are directly used for gene expression stability comparison. From the results shown in Figure 1, eight gene regions (*bTUB*_utr3, *HIS*_utr, *EF-1a*_utr2, *GAP*_utr2, *ACT*_utr1, *ANX*_utr, *SAND*_utr and *TIP*_utr) were selected from each homologous gene groups based on a tendency of much narrow range distribution of *Ct* values. Mean *Ct* values were calculated in each run for each gene/samples. The differences of *Ct* values (ΔCt) were calculated for each pair-wise gene combinations and their distributions were evaluated by standard deviation of ΔCt [18].

## Supporting Information

Figure S1 **Direct sequencing of amplified fragments generated with RT-qPCR primers.** Fragments amplified with each RT-qPCR primers (Table S4) were directly sequenced by ABI 3130xl Genetic Analyzer. Chromatograms are trimmed by manually to remove low quality sequences. Alignment was done by Geneious 5.0.2 (Biomatters Ltd.). Sequences trimmed from 3′-RACE clones are indicated on top, sequences of amplified fragments with RT-qPCR primers are shown lower two lines. For *SAND*_utr region, sequences of genomic fragments obtained from genomic DNA template (Figure S2, lane g) were also shown. Note that *Ex Taq* polymerase add nucleotide “A” at the 3′-end of the amplicon, the last nucleotide of forward direction is always “A”, and the first nucleotide of reversed sequence is always “T” (corresponding to the last nucleotide “A” in reverse direction).
(DOC)

Figure S2 **Confirmation of no genomic DNA contamination in RNA samples used.** RNA samples are treated with (indicated by “+”) or without (by “–”) reverse transcriptase (RT) followed by RNaseH. The resultant mixtures are one tenth diluted and 0.8 µl aliquot was amplified with *SAND*_utr primers (Table S4). 1: FB, Sep. 30; 2: FB, Dec. 15; 3: FO, Petals; 4: FO, Anthers; 5: FS, Jul. 3; 6: FS, Sep. 3; 7: FF, May 22; 8: FF Aug. 12. M: marker DNAs. g: genomic DNA template (positive control). Amplified fragments from gDNA (1.2 kb) and cDNA (0.15 kb) are indicated by arrows.
(TIF)

Table S1 **List of known functions of reference gene candidates.**
(XLS)

Table S2 **Amino acid and nucleotide identity among homologous genes tested in the study.** Identities between homologous genes are presented. For deduced amino acid residues, the length of the residues compared are also shown after the slash. Nucleotide identities are compared in CDS regions and also in the first 150 nucleotides in 3′-UTR regions.
(XLS)

Table S3 **Amino acid of deduced protein (A) or nucleotide (3′-UTR only) (B) identity of cold-inducible genes CBF2 and CBF4 in apple and Japanese pear.**
(XLS)

Table S4 **List of primers used for RT-qPCR.**
(XLS)
