# Supplementary material for: Evaluation of Reference Genes for Accurate Normalization of Gene Expression for Real Time-Quantitative PCR in Pyrus pyrifolia Using Different Tissue Samples and Seasonal Conditions
Source: PLoS One. 2014 Jan 22;9(1):e86492. doi: 10.1371/journal.pone.0086492 (PMC3899261; doi:10.1371/journal.pone.0086492)
Supplement: Figure S1 — Direct sequencing of amplified fragments generated with RT-qPCR primers. Fragments amplified with each RT-qPCR primers (Table S4) were directly sequenced by ABI 3130xl Genetic Analyzer. Chromatograms are trimmed by manually to remove low quality sequences. Alignment was done by Geneious 5.0.2 (Biomatters Ltd.). Sequences trimmed from 3′-RACE clones are indicated on top, sequences of amplified fragments with RT-qPCR primers are shown lower two lines. For SAND_utr region, sequences of genomic fragments obtained from genomic DNA template (Figure S2, lane g) were also shown. Note that Ex Taq polymerase add nucleotide “A” at the 3′-end of the amplicon, the last nucleotide of forward direction is always “A”, and the first nucleotide of reversed sequence is always “T” (corresponding to the last nucleotide “A” in reverse direction). (DOC) [file pone.0086492.s001.doc]

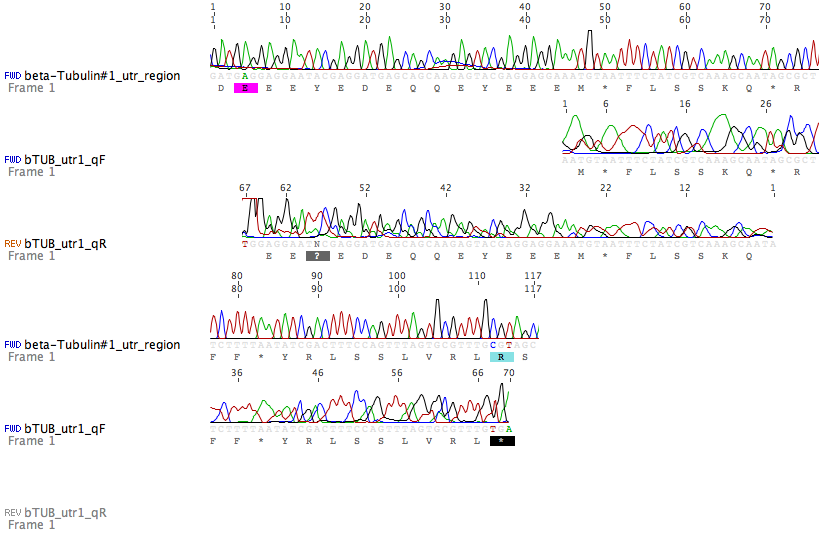
**A**

**Figure S1A Direct sequence confirmation of amplified fragment by *bTUB*_utr1 primers.** Alignment was done by Geneious 5.0.2. Unless mentioned 3’-RACE clones are isolated from cultivar “Kosui”. beta-Tubulin#1_utr_region: Part of the sequence from 3’-RACE clone *beta-Tubulin#1*. bTUB_utr1_qF: Sequence of amplified fragment by *bTUB*_utr1_qF primer. bTUB_utr1_qR: Sequence of amplified fragment by *bTUB*_utr1_qR primer.

**B**

**
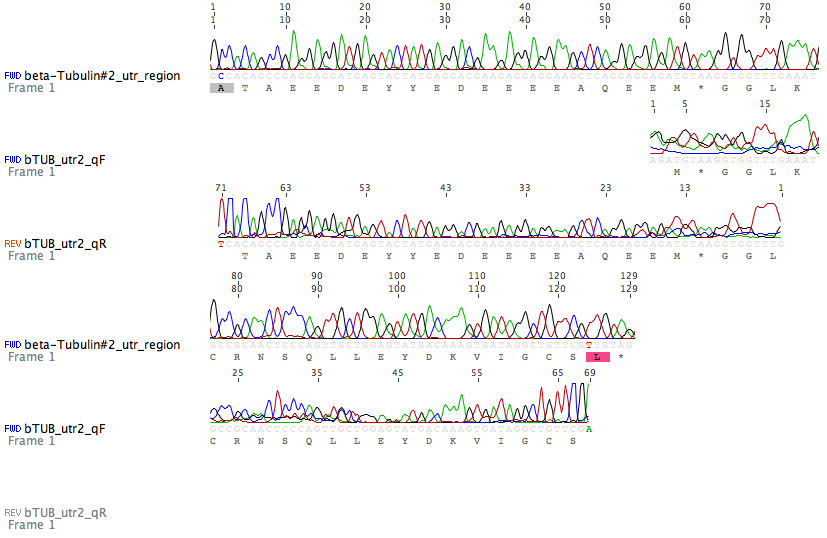
**

**Figure S1B Direct sequence confirmation of amplified fragment by *bTUB*_utr2 primers.** beta-Tubulin#2_utr

_region: Part of the sequence from 3’-RACE clone *beta-Tubulin#2*. bTUB_utr2_qF: Sequence of amplified fragment by *bTUB*_utr2_qF primer. bTUB_utr2_qR: Sequence of amplified fragment by *bTUB*_utr2_qR primer.

**C**

**
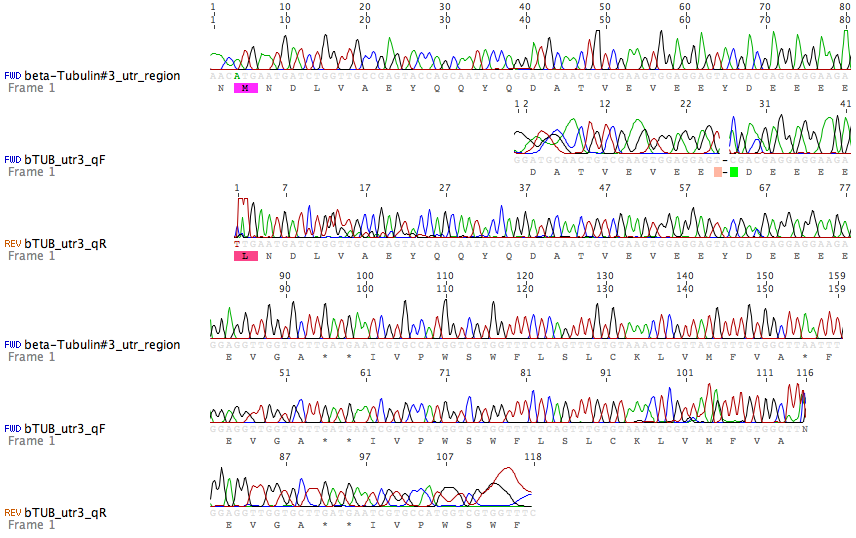
**

**Figure S1C Direct sequence confirmation of amplified fragment by *bTUB*_utr3 primers.** beta-Tubulin#3_utr

_region: Part of the sequence from 3’-RACE clone *beta-Tubulin#3*. bTUB_utr3_qF: Sequence of amplified fragment by *bTUB*_utr3_qF primer. bTUB_utr3_qR: Sequence of amplified fragment by *bTUB*_utr3_qR primer.

**D**

**
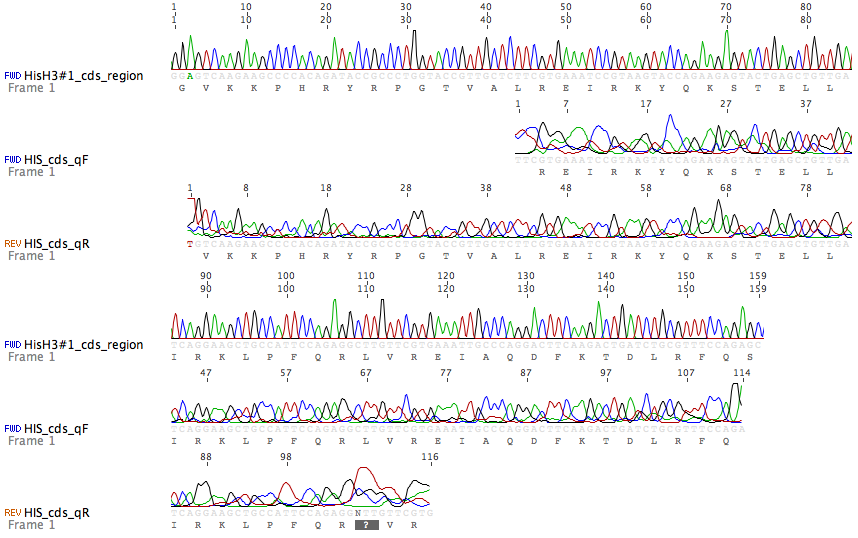
**

**Figure S1D Direct sequence confirmation of amplified fragment by *HIS*_cds primers.** HisH3#1_cds_region: Part of the sequence from 3’-RACE clone *HistoneH3#1*. HIS_cds_qF: Sequence of amplified fragment by *HIS*_cds_qF primer. HIS_cds_qR: Sequence of amplified fragment by *HIS*_cds_qR primer.

**E**

**
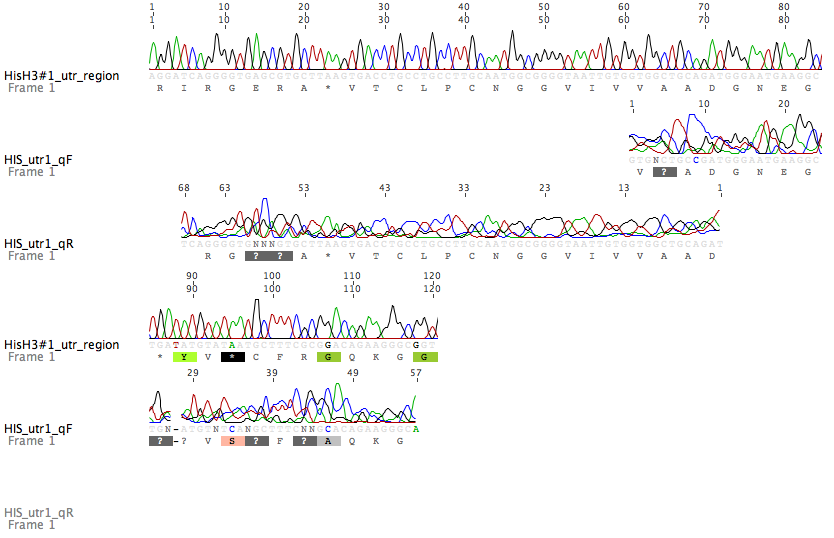
**

**Figure S1E Direct sequence confirmation of amplified fragment by *HIS*_utr1 primers.** HisH3#1_utr_region: Part of the sequence from 3’-RACE clone *HistoneH3#1*. HIS_utr1_qF: Sequence of amplified fragment by *HIS*_utr1_qF primer. HIS_utr1_qR: Sequence of amplified fragment by *HIS*_utr1_qR primer.

**F**

**
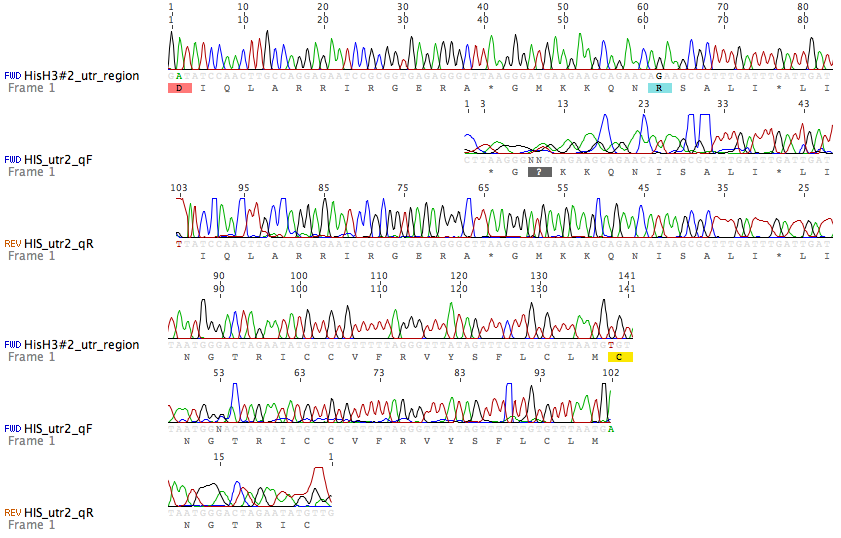
**

**Figure S1F Direct sequence confirmation of amplified fragment by *HIS*_utr2 primers.** HisH3#2_utr_region: Part of the sequence from 3’-RACE clone *HistoneH3#2* isolated from ‘TP-85-119’ line. HIS_utr2_qF: Sequence of amplified fragment from cultivar ‘Kosui’ by *HIS*_utr2_qF primer. HIS_utr2_qR: Sequence of amplified fragment by *HIS*_utr2_qR primer. Note that change at 62th nucleotide G to T is probably due to single nucleotide polymorphism between the lines.

**G**

**
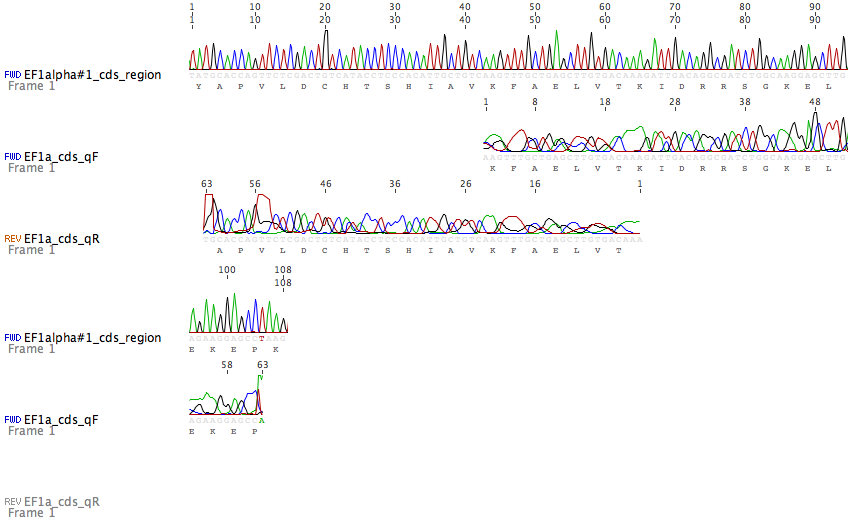
**

**Figure S1G Direct sequence confirmation of amplified fragment by *EF1a*_cds primers.** EF1alpha#1_cds_region: Part of the sequence from 3’-RACE clone *EF-1alpha#1*. EF1a_cds_qF: Sequence of amplified fragment by *EF1a*_cds_qF primer. EF1a_cds_qR: Sequence of amplified fragment by *EF1a*_cds_qR primer.

**H**

**
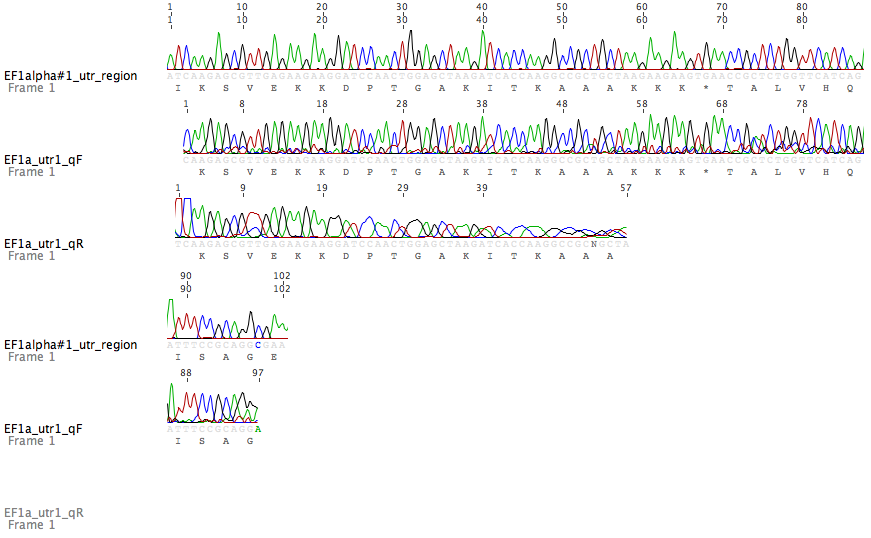
**

**Figure S1H Direct sequence confirmation of amplified fragment by *EF1a*_utr1 primers.** EF1alpha#1_utr_region: Part of the sequence from 3’-RACE clone *EF-1alpha#1*. EF1a_utr1_qF: Sequence of amplified fragment by *EF1a*_utr1_qF primer. EF1a_utr1_qR: Sequence of amplified fragment by *EF1a*_utr1_qR primer.

**I**

**
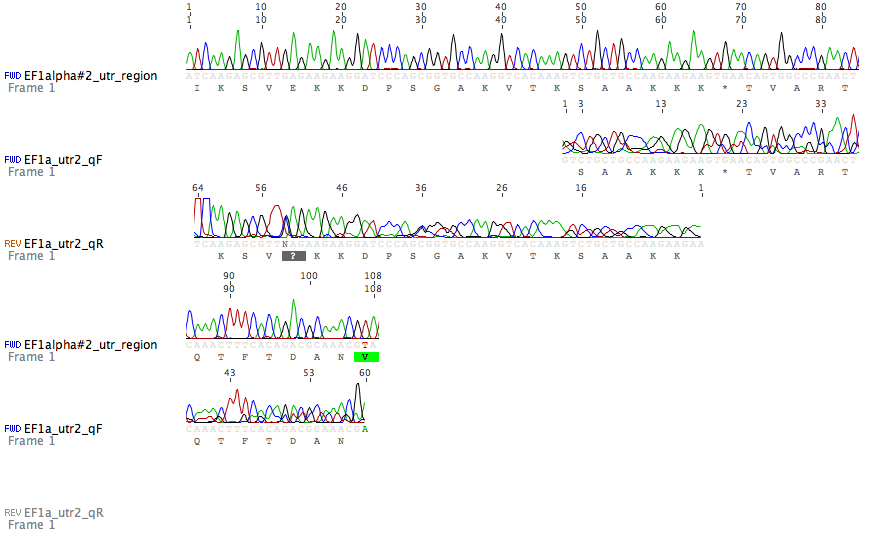
**

**Figure S1I Direct sequence confirmation of amplified fragment by *EF1a*_utr2 primers.** EF1alpha#2_utr_region: Part of the sequence from 3’-RACE clone *EF-1alpha#2*. EF1a_utr2_qF: Sequence of amplified fragment by *EF1a*_utr2_qF primer. EF1a_utr2_qR: Sequence of amplified fragment by *EF1a*_utr2_qR primer.

**J**

**
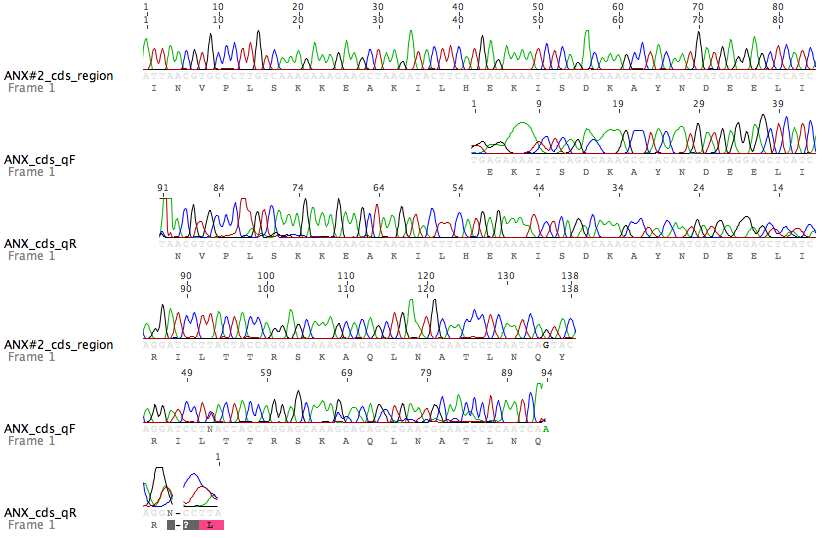
**

**Figure S1J Direct sequence confirmation of amplified fragment by *ANX*_cds primers.** ANX#2_cds_region: Part of the sequence from 3’-RACE clone *Annexin#2*. ANX_cds_qF: Sequence of amplified fragment by *ANX*_cds_qF primer. ANX_cds_qR: Sequence of amplified fragment by *ANX*_cds_qR primer.

**K**

**
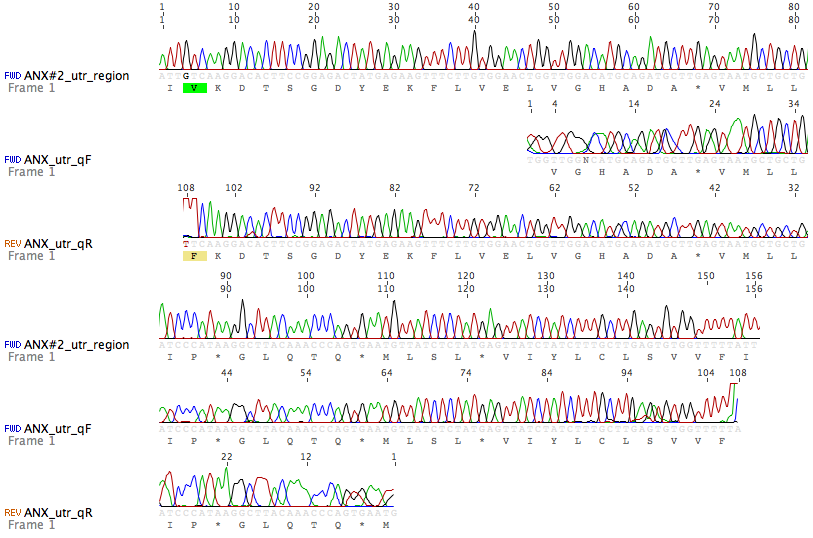
**

**Figure S1K Direct sequence confirmation of amplified fragment by *ANX*_utr primers.** ANX#2_utr_region: Part of the sequence from 3’-RACE clone *Annexin#2*. ANX_utr_qF: Sequence of amplified fragment by *ANX*_utr_qF primer. ANX_utr_qR: Sequence of amplified fragment by *ANX*_utr_qR primer.


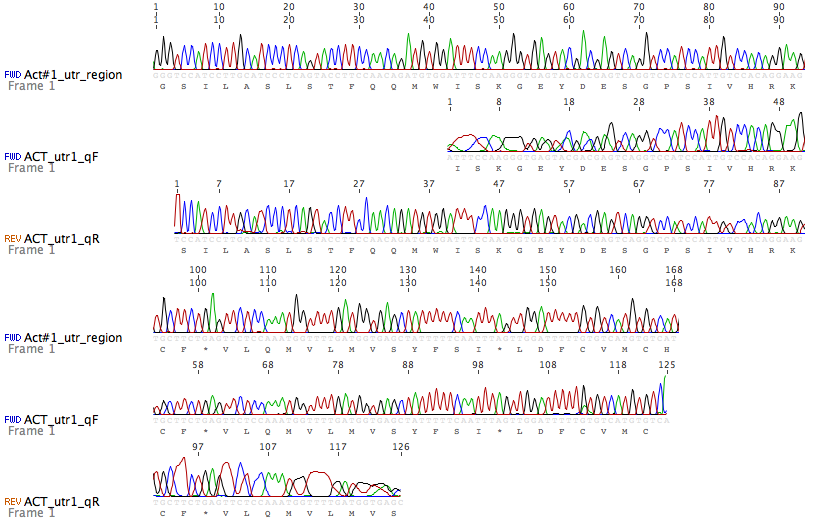
**L**

**Figure S1L Direct sequence confirmation of amplified fragment by *ACT*_utr1 primers.** Act#1_utr_region: Part of the sequence from 3’-RACE clone *Actin#1*. ACT_utr1_qF: Sequence of amplified fragment by *ACT*_utr1_qF primer. ACT_utr1_qR: Sequence of amplified fragment by *ACT*_utr1_qR primer.

**M**

**
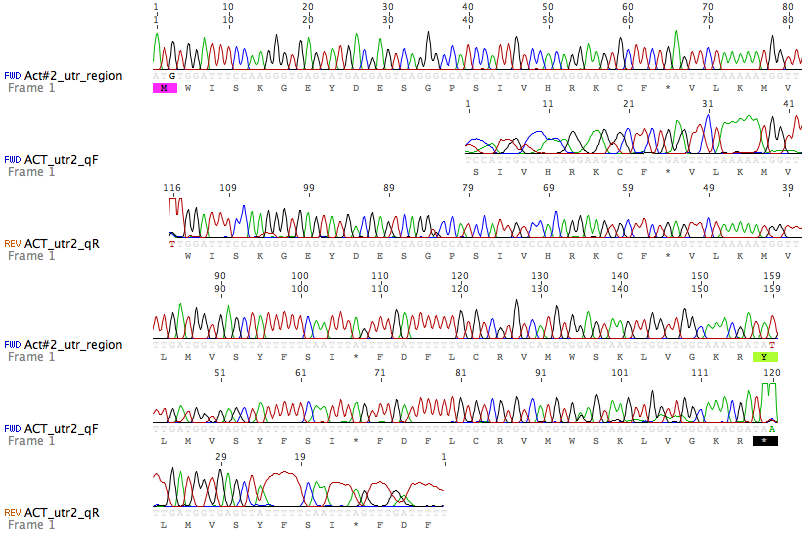
**

**Figure S1M Direct sequence confirmation of amplified fragment by *ACT*_utr2 primers.** Act#2_utr_region: Part of the sequence from 3’-RACE clone *Actin#2*. ACT_utr2_qF: Sequence of amplified fragment by *ACT*_utr2_qF primer. ACT_utr2_qR: Sequence of amplified fragment by *ACT*_utr2_qR primer.

**N**

**
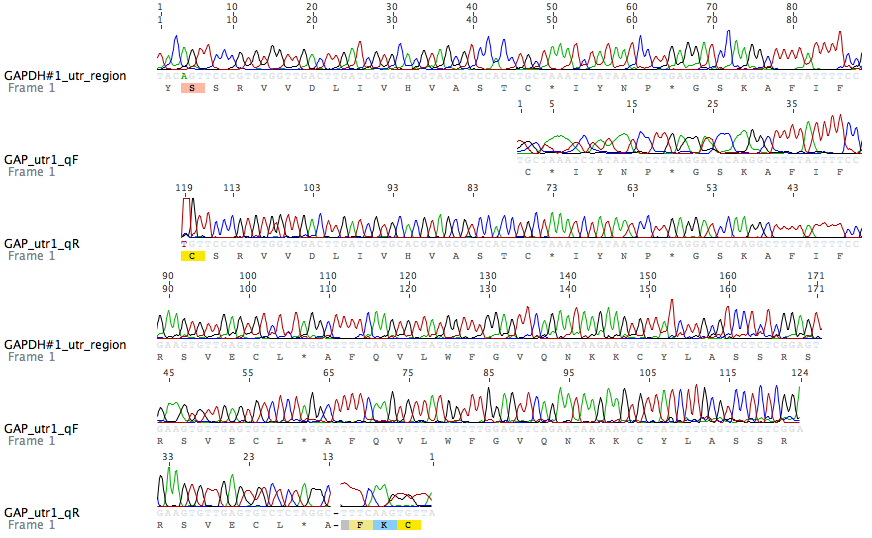
**

**Figure S1N Direct sequence confirmation of amplified fragment by *GAP*_utr1 primers.** GAPDH#1_utr_region: Part of the sequence from 3’-RACE clone *GAPDH#1*. GAP_utr1_qF: Sequence of amplified fragment by G*AP*_utr1_qF primer. GAP_utr1_qR: Sequence of amplified fragment by *GAP*_utr1_qR primer.

**O**

**
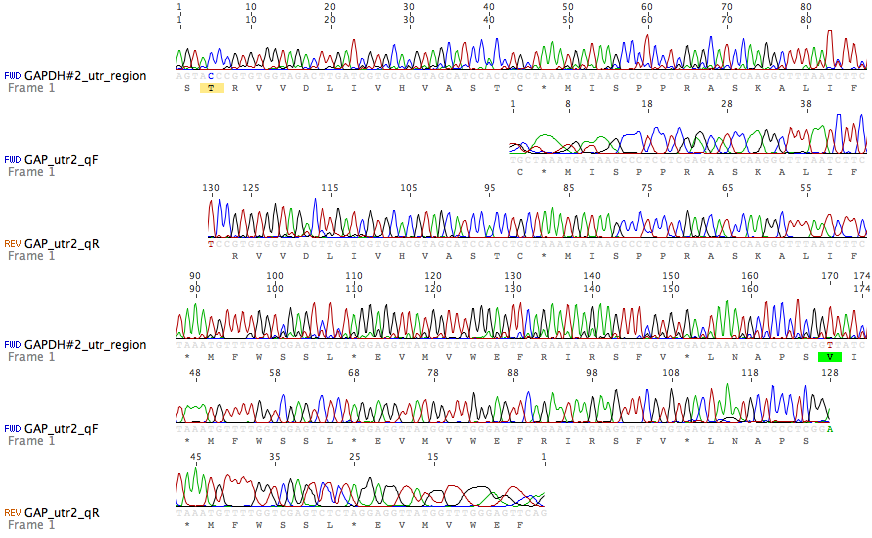
**

**Figure S1O Direct sequence confirmation of amplified fragment by *GAP*_utr2 primers.** GAPDH#2_utr_region: Part of the sequence from 3’-RACE clone *GAPDH#2*. GAP_utr2_qF: Sequence of amplified fragment by G*AP*_utr2_qF primer. GAP_utr2_qR: Sequence of amplified fragment by *GAP*_utr2_qR primer.

**P**

**
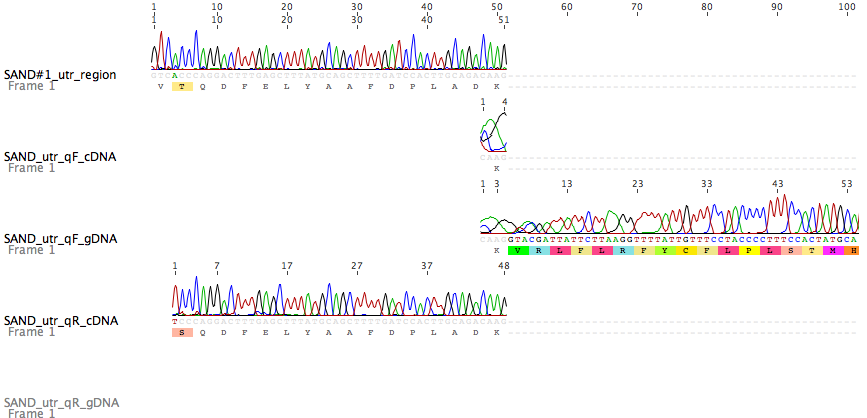
**

**Figure S1P Direct sequence confirmation of amplified fragment by *SAND*_utr primers.** SAND#1_utr_region: Part of the sequence from 3’-RACE clone *SAND#1*. SAND_utr_qF: Sequence of amplified fragment by *SAND*_utr_qF primer. SAND_utr_qR: Sequence of amplified fragment by *SAND*_utr_qR primer. Template DNA (cDNA or gDNA) was shown after the primer name. It was revealed that *SAND* gene has an 1155 bp-long intron between the primer sites corresponding to *SAND*_utr_qF and *SAND*_utr_qR. Pothograph of DNA fragments on agarose gel is also shown in Figure S2.

**
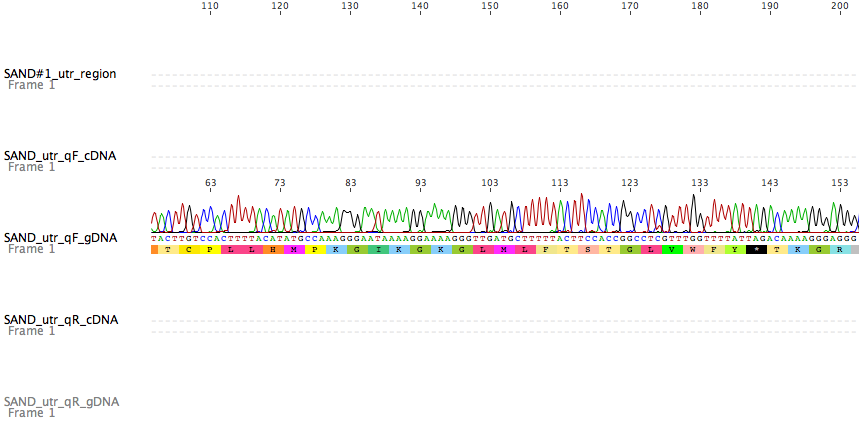
**

**Figure S1P** Continued.

**
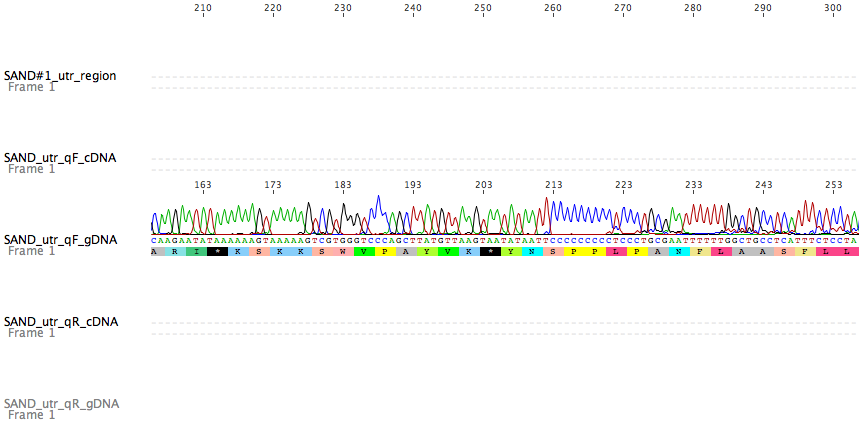
**

**Figure S1P** Continued.

**
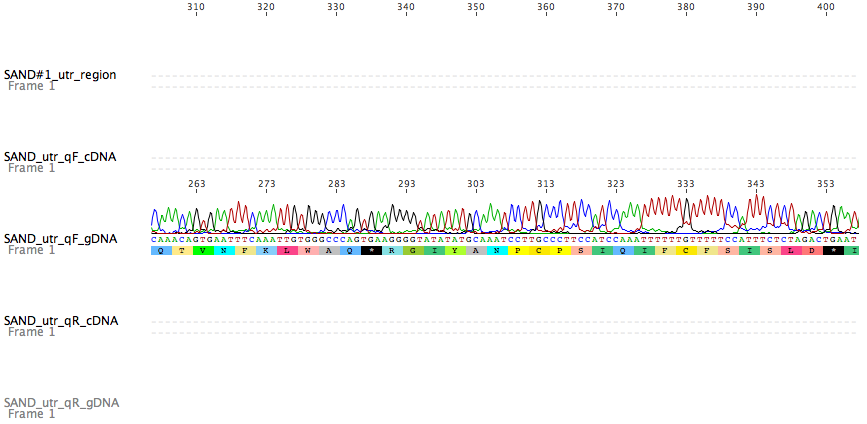
**

**Figure S1P** Continued.

**
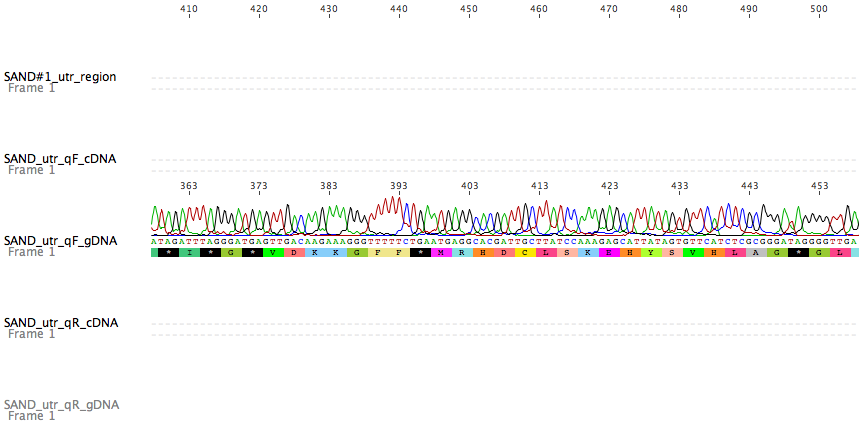
**

**Figure S1P** Continued.

**
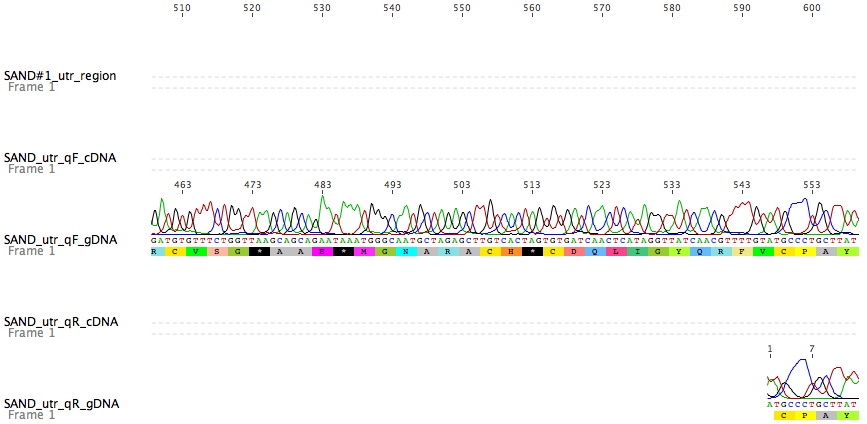
**

**Figure S1P** Continued.

**
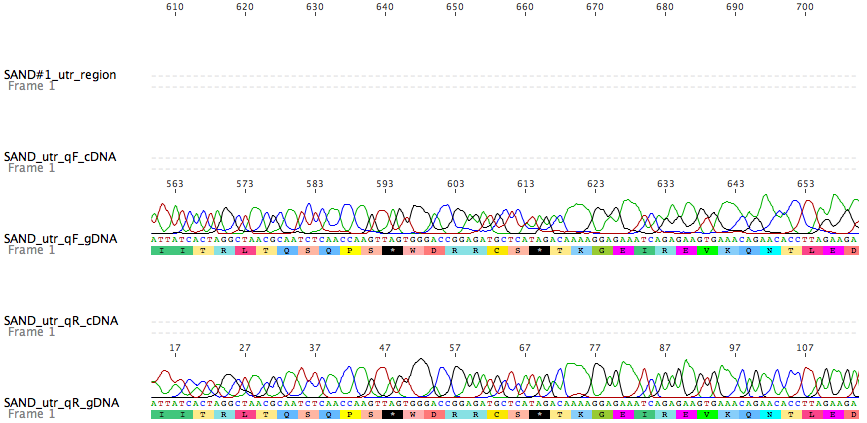
**

**Figure S1P** Continued.

**
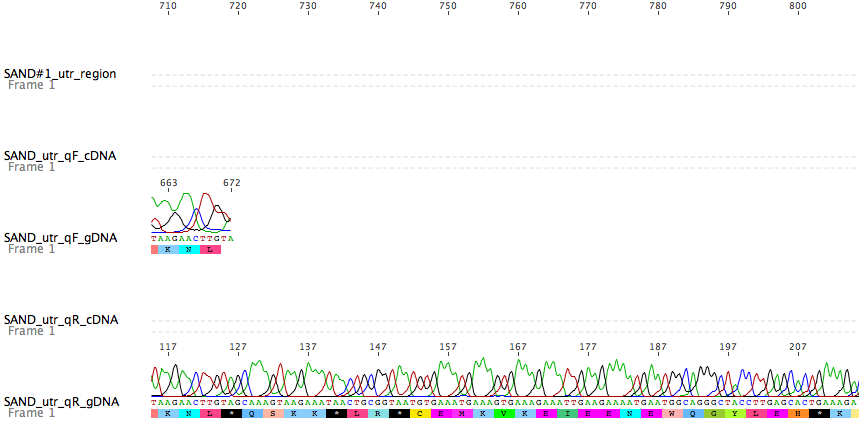
**

**Figure S1P** Continued.

**
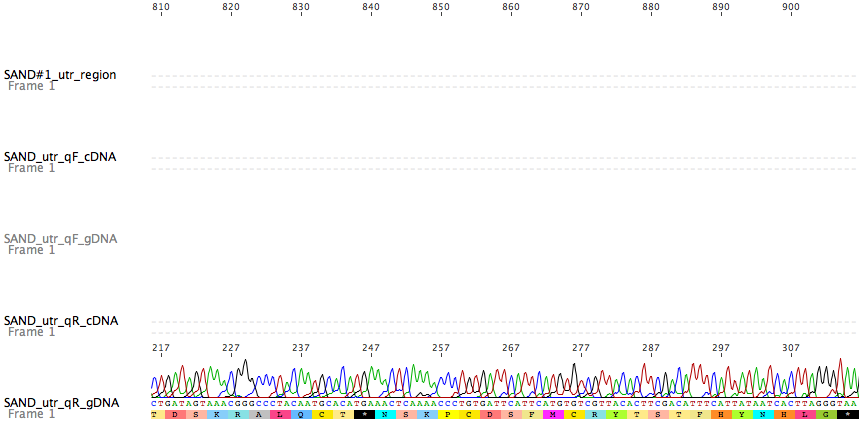
**

**Figure S1P** Continued.

**
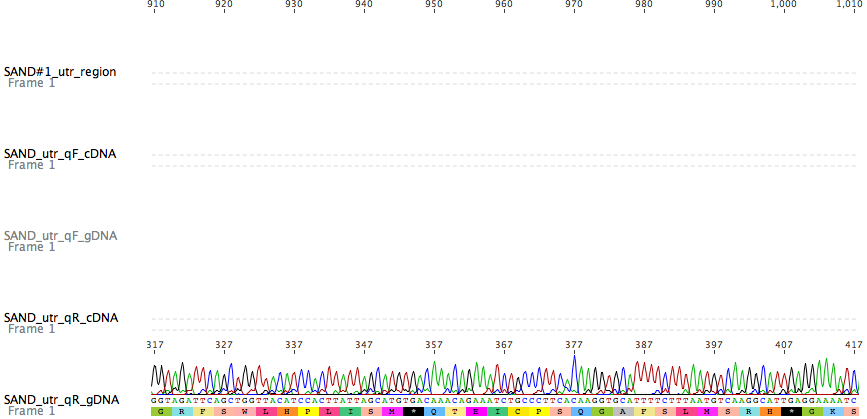
**

**Figure S1P** Continued.

**
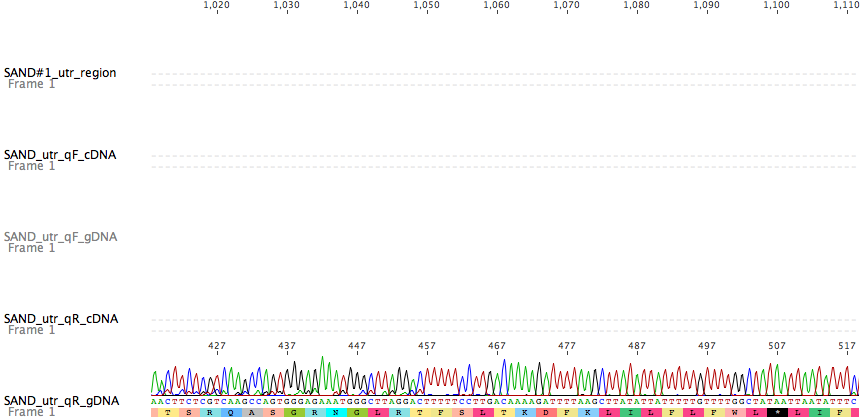
**

**Figure S1P** Continued.

**
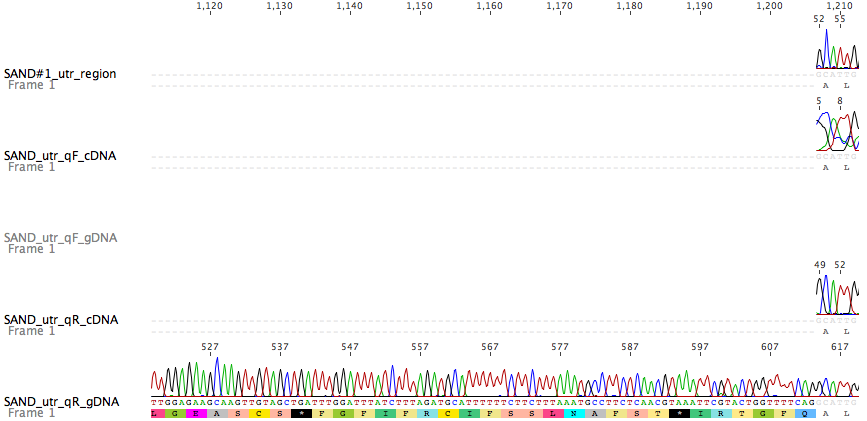
**

**Figure S1P** Continued.

**
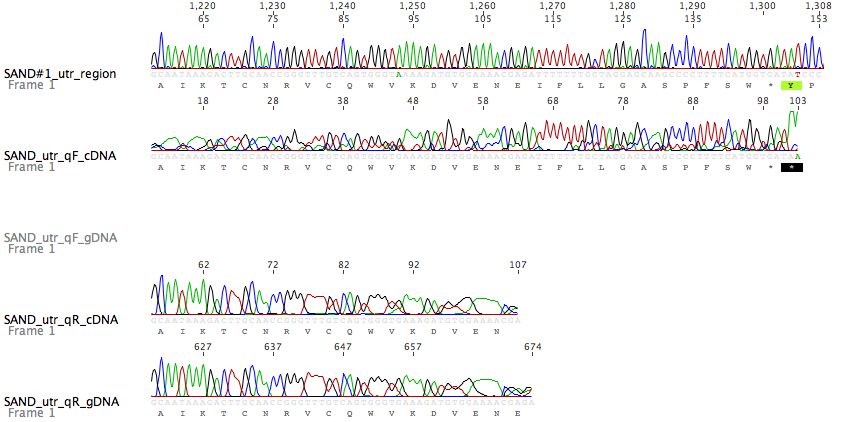
**

**Figure S1P** Continued.

**Q**

**
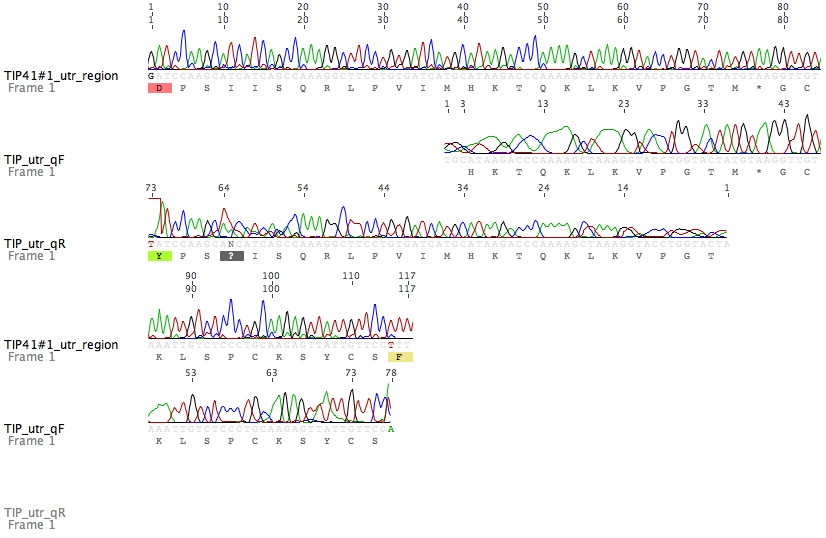
**

**Figure S1Q Direct sequence confirmation of amplified fragment by *TIP*_utr primers.** TIP41#1_utr_region: Part of the sequence from 3’-RACE clone *TIP41#1*. TIP_utr_qF: Sequence of amplified fragment by *TIP*_utr_qF primer. TIP_utr_qR: Sequence of amplified fragment by *TIP*_utr_qR primer.


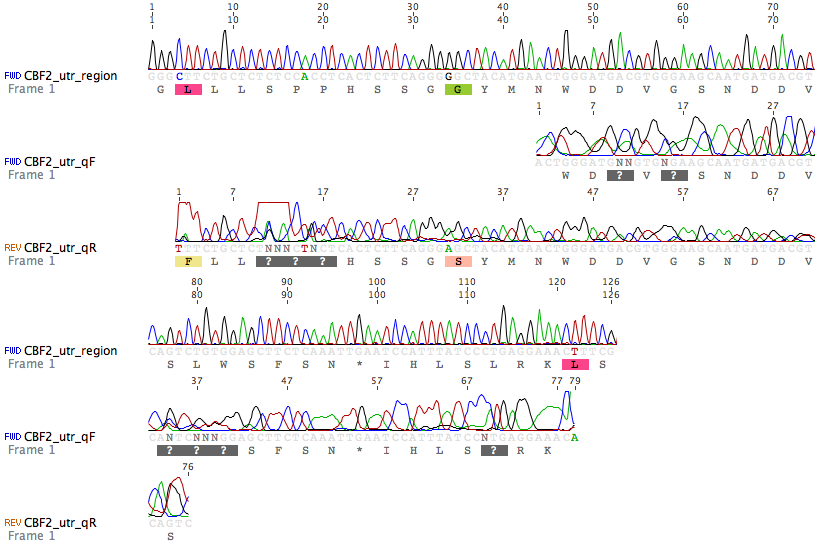
**R**

**Figure S1R Direct sequence confirmation of amplified fragment by *CBF2*_utr primers.** CBF2_utr_region: Part of the sequence from 3’-RACE clone *CBF#1412*. CBF2_utr_qF: Sequence of amplified fragment by *CBF2*_utr_qF primer. CBF2_utr_qR: Sequence of amplified fragment by *CBF2*_utr_qR primer.

**S**

**
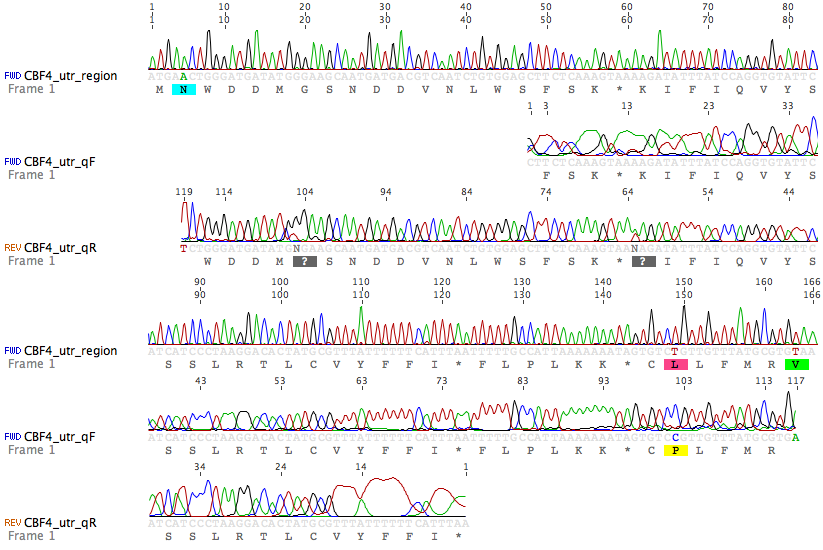
**

**Figure S1S Direct sequence confirmation of amplified fragment by *CBF4*_utr primers.** CBF4_utr_region: Part of the sequence from 3’-RACE clone *CBF#1403*. CBF4_utr_qF: Sequence of amplified fragment by *CBF4*_utr_qF primer. CBF4_utr_qR: Sequence of amplified fragment by *CBF4*_utr_qR primer.
