# Supplementary figures and images for: Evaluation of Reference Genes for Accurate Normalization of Gene Expression for Real Time-Quantitative PCR in Pyrus pyrifolia Using Different Tissue Samples and Seasonal Conditions
Source: PLoS One. 2014 Jan 22;9(1):e86492. doi: 10.1371/journal.pone.0086492 (PMC3899261; doi:10.1371/journal.pone.0086492)

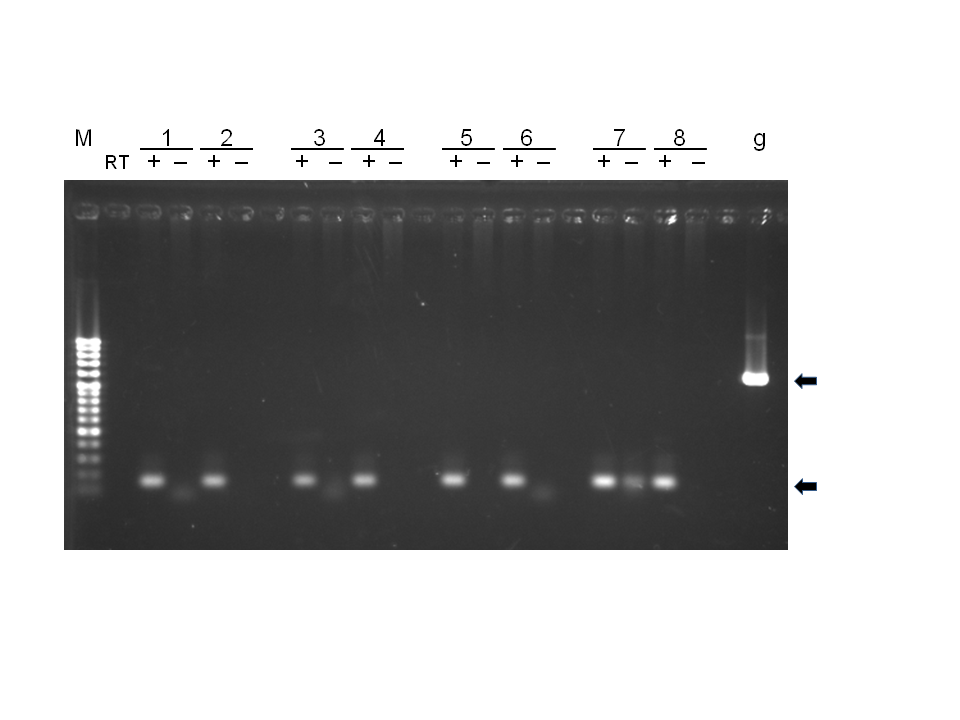

Supplement: Figure S2 — Confirmation of no genomic DNA contamination in RNA samples used. RNA samples are treated with (indicated by “+”) or without (by “–”) reverse transcriptase (RT) followed by RNaseH. The resultant mixtures are one tenth diluted and 0.8 µl aliquot was amplified with SAND_utr primers (Table S4). 1: FB, Sep. 30; 2: FB, Dec. 15; 3: FO, Petals; 4: FO, Anthers; 5: FS, Jul. 3; 6: FS, Sep. 3; 7: FF, May 22; 8: FF Aug. 12. M: marker DNAs. g: genomic DNA template (positive control). Amplified fragments from gDNA (1.2 kb) and cDNA (0.15 kb) are indicated by arrows. (TIF) [file pone.0086492.s002.tif]
